# Efficacy and Safety of mRNA-Based COVID-19 Vaccines in Solid Organ Transplant Recipients: A Systematic Review and Meta-Analysis

**DOI:** 10.3390/vaccines14010072

**Published:** 2026-01-08

**Authors:** Maya Alkhidir, Kannan Sridharan

**Affiliations:** 1 College of Medicine & Health Sciences, Arabian Gulf University, Manama 26671, Bahrain; mayamok@agu.edu.bh; 2 Department of Pharmacology & Therapeutics, College of Medicine & Health Sciences, Arabian Gulf University, Manama 26671, Bahrain

**Keywords:** mRNA vaccine, organ transplantation, immune responsiveness, seroconversion

## Abstract

Background: Solid organ transplant recipients (SOTRs) are highly vulnerable to severe COVID-19 infection, yet initial vaccine trials provided limited data on efficacy and safety in this immunocompromised population. Heterogeneous seroconversion rates and conflicting safety reports complicate the formulation of clear clinical guidelines. This systematic review and meta-analysis aim to aggregate existing evidence to determine the precise seroconversion and safety profiles of COVID-19 vaccines and identify key factors influencing immune response in SOTRs. Methods: A comprehensive literature search was conducted identifying 125 studies evaluating WHO/FDA-authorized vaccines in SOTRs. Outcomes were the pooled seroconversion proportion and safety profile. Subgroup analyses were performed based on vaccine type, transplanted organ, number of doses, and prior SARS-CoV-2 infection status, confirmed by leave-one-out sensitivity analysis and bootstrap methods. Results: Most studies assessed mRNA-based vaccines (123/125, 98.4%). The overall pooled seroconversion proportion across all SOTRs was significantly blunted at 0.49 (95% CI, 0.43 to 0.55), demonstrating high heterogeneity (I^2^ = 94.2%). Seroconversion showed a clear positive dose–response relationship, increasing from 27% after one dose to 84% after four doses. Prior COVID-19 infection was the strongest predictor of a response, resulting in a pooled seroconversion of 0.90 (95% CI, 0.82 to 0.94; I^2^ = 0%). Organ-specific analyses revealed the highest response in Liver recipients (0.80) and the lowest in Lung recipients (0.29). Vaccine platform analysis showed that the highest response was with mRNA-1273 (0.55) and the lowest with CoronaVac (0.29). The safety profile was limited. Conclusions: SOTRs exhibit profound hypo responsiveness to COVID-19 vaccines; however, the extreme heterogeneity observed across studies necessitates a cautious interpretation of pooled seroconversion estimates. While the data indicates a significant dose–response relationship favoring an aggressive, multi-dose strategy, the apparent safety profile may reflect under-reporting and limited follow-up rather than confirmed safety equivalence. Rare but clinically critical outcomes, such as acute allograft rejection, remain inadequately characterized in the current literature. Consequently, while the prioritization of multi-dose regimens and hybrid immunity is supported to maximize protection, clinicians must recognize that individual responses remain highly variable, and the long-term immunological impact of repeated stimulation requires further standardized investigation.

## 1. Introduction

The COVID-19 pandemic, caused by the severe acute respiratory syndrome coronavirus 2 (SARS-CoV-2), was officially declared by the World Health Organization (WHO) on 11 March 2020, following the elevation of its global risk assessment to the highest level of concern on 21 February 2020. As of 2025, the global crisis has resulted in an estimated 7 million deaths and over 770 million confirmed cases worldwide, highlighting its devastating impact on global public health and healthcare infrastructure [1]. The scale of the crisis spurred an unprecedented and rapid international response. Within weeks of the pandemic declaration, by 8 April 2020, 115 vaccine candidates were already in preclinical development, with five having progressed to phase 1 clinical trials. Accelerated regulatory authorizations from agencies like the U.S. Food and Drug Administration (FDA) and the European Medicines Agency (EMA) facilitated the rapid implementation of mass immunization programs globally [2].

A remarkable achievement was the regulatory approval of the first COVID-19 vaccines less than 10 months after phase 1 trials commenced. This represented a dramatic acceleration compared to the typical pre-pandemic timeline for vaccine development, which often spanned more than a decade due to the sequential nature of preclinical studies, phased clinical trials, regulatory review, manufacturing, quality assurance, and post-marketing surveillance [1]. Extensive global cooperation underpinned the urgent need for vaccination. In 2020, the White House COVID-19 Response Team in the United States supported the development of six vaccine platforms: messenger RNA (mRNA) technologies (Pfizer/BioNTech and Moderna), viral vector technologies (Johnson & Johnson/Janssen and AstraZeneca/Oxford), and protein subunit vaccines (Novavax and Sanofi/GSK) [3].

The Pfizer/BioNTech BNT162b2 vaccine, an mRNA vaccine encapsulated in lipid nanoparticles that encodes the SARS-CoV-2 spike protein, was the first to receive Emergency Use Authorization (EUA) in the U.S. on 11 December 2020 and subsequently gained full approval on 23 August 2021. It demonstrated 95% efficacy against moderate to severe COVID-19 when administered as a two-dose regimen 21 days apart, by facilitating antigen expression and inducing protective immunity [3]. Moderna’s mRNA-1273 was the second vaccine to receive EUA (17 December 2020). It showed 94% efficacy against severe disease and is administered as two doses 28 days apart. Preclinical studies demonstrated robust, dose-dependent antibody responses. Common adverse effects associated with both mRNA vaccines, such as injection site pain, headache, and lymphadenopathy, are reported to occur more frequently after the second dose [3]. Johnson & Johnson’s Ad26.COV2.S, a single-dose adenoviral vector vaccine, was first approved by the FDA for EUA on 27 February 2021. It demonstrated 66.9% efficacy and elicited strong neutralizing antibody responses in preclinical studies [3], although its use was briefly paused for safety concerns and resumed in May 2021. Novavax, a protein subunit vaccine approved for EUA on 13 July 2022, showed 90% efficacy as a two-dose series administered 21 days apart, with a side effect profile like that of the mRNA vaccines. Both the Moderna and Pfizer vaccines have since received approval for booster doses, and their successful development has been pivotal in the global effort to control SARS-CoV-2 transmission and disease severity [3].

Immunocompromised patients are defined as individuals with deficient immunologic mechanisms due to an underlying disorder, another disease, or the administration of immunosuppressive drugs or radiation therapy [4]. They were typically excluded from initial vaccine trials, resulting in limited data on vaccine efficacy and safety within this population. Constituting approximately 3% of the population, these adults are of particular concern due to their increased risk for severe infection, prolonged viral shedding, and the potential for their underlying conditions or ongoing treatments (which may involve immune system overactivation or suppression) to interfere with vaccine effectiveness [5]. Consequently, reliable data on vaccine efficacy and safety are urgently needed for this population [5]. Among immunocompromised populations, solid organ transplant recipients (SOTRs) represent a particularly vulnerable subgroup [6]. SOTRs face unique clinical challenges, including high susceptibility to opportunistic infections, rapid disease progression, elevated mortality risk from COVID-19, difficulties in managing immunosuppressive regimens with limited evidence, potential drug–drug interactions, and the concurrent short- and long-term risks of allograft rejection or loss [6].

While COVID-19 vaccines have demonstrated strong seroconversion and favorable safety profiles in the general population, the data regarding their efficacy and safety in SOTRs remain heterogeneous and inconclusive [6,7,8,9,10,11,12,13]. Some studies report satisfactory seroconversion rates in this cohort, while others document significantly diminished immune responses, raising concerns about suboptimal protection against SARS-CoV-2 [6,7,8,9,10,11,12,13]. Similarly, the safety profile is variably reported, with some studies describing good tolerability and others documenting episodes of graft rejection or immunological complications post-vaccination [6,7,8,9,10,11,12,13]. This considerable variability complicates the formulation of clear, evidence-based clinical guidelines for the vaccination of transplant recipients. Consequently, a comprehensive systematic review and meta-analysis are warranted to systematically aggregate the existing literature, yield more precise estimates of vaccine-associated seroconversion and safety outcomes, and thoroughly elucidate the factors contributing to the observed heterogeneity. Such an analysis is critical for addressing existing knowledge gaps, refining vaccination strategies, and informing targeted preventive measures for this vulnerable population.

## 2. Methods

### 2.1. Search Methods

The protocol for this ancillary systematic review was prospectively registered in the Open Science Framework [14]. A comprehensive literature search was conducted across three electronic databases: Medline (via PubMed), Cochrane Central Register of Controlled Trials (CENTRAL), and Google Scholar, to identify eligible studies that evaluated the efficacy and safety of COVID-19 vaccines in organ transplant patients. Details of the search strategy are summarized in the Electronic Supplementary Table S1. The search was last updated on 14 January 2025. We did not place any restrictions regarding the language or year of the publications. We excluded conference abstracts, reviews and editorials. References from the eligible articles were examined and references pertinent to this study were included.

### 2.2. Eligibility Criteria

○ Population: We considered studies that included participants who underwent any solid organ transplantation such as kidney, liver, lung, heart including multiple organ transplantation.
○ Intervention: Administration of any WHO- or FDA-authorized COVID-19 vaccine that includes the following were considered in this study: BNT162b2 Pfizer-BioNTech, mRNA-1273 Moderna, AZD1222 Oxford-AstraZeneca, Ad26.COV2.S Janssen/Johnson & Johnson, NVX-CoV2373 Novavax, inactivated vaccines. We included studies that evaluated both the primary dosing regimens as well as the booster doses.
○ Comparator: Any of the above vaccines/placebo/studies with single vaccine arm.
○ Outcomes:
○ Seroconversion rate: Proportion of patients with antibodies above a pre-defined threshold) as defined in each of the studies included. In this study, seroconversion has been considered as a proxy marker of humoral immunogenicity.
○ Safety: Proportion of patients reporting local and systemic adverse events.
○ Study Designs: We included studies with any comparative design such as randomized controlled trials (RCTs), non-randomized interventional studies, and observational studies such as prospective and retrospective cohort, and case–control designs.

### 2.3. Study Selection Process

Two authors independently were involved in screening the articles for their suitability to be included. The full texts of potentially relevant articles were retrieved and independently assessed for eligibility and the following details were extracted: study characteristics (first author, year, country, design, setting, follow-up period); population (sample size, specific organ transplantation), age, sex, comorbidities; intervention (vaccine name, number of doses, timing of doses); and outcome data (definition of seroconversion, number of events and total participants in each group, and safety assessment). The risk of bias of included studies was assessed using Cochrane tool for RCTs and Joanna Briggs Institute (JBI) tool for other study designs [15,16]. Statistical analysis was carried out using R (version 4.5.1). Publication biases were assessed using Egger’s regression analyses. Single pooled proportion using random-effects modeling was estimated for seroconversion rate across studies. The following subgroup analyses were carried out: vaccine types, type of transplanted organs, number of vaccine doses and prior COVID-19 infection. Leave-one-out sensitivity analyses were conducted to assess the robustness of findings by excluding studies one by one and assessing their impact on the pooled estimates. Bootstrap analyses with 1000 iterations were carried out to improve the robustness of the findings.

## 3. Results

### 3.1. Search Results

**Figure 1:**
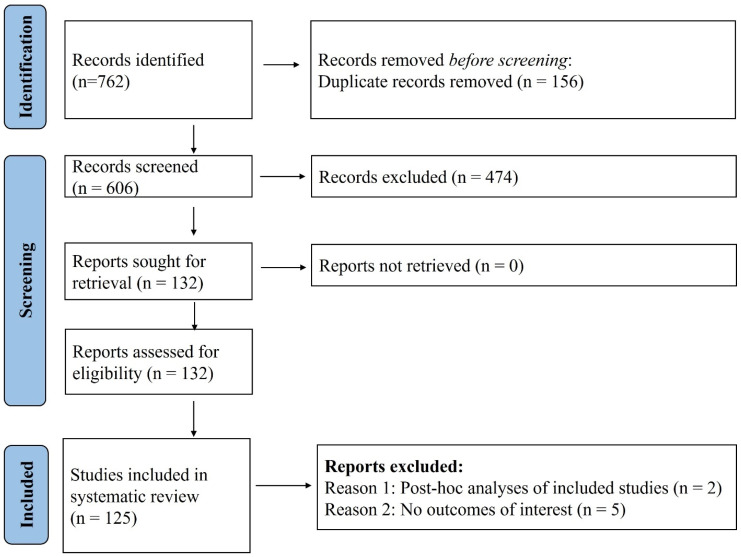
PRISMA flow diagram. A total of 125 studies were included in the systematic review out of the total 762 obtained with the search strategy.

This systematic review and meta-analysis included a total of 125 studies [17,18,19,20,21,22,23,24,25,26,27,28,29,30,31,32,33,34,35,36,37,38,39,40,41,42,43,44,45,46,47,48,49,50,51,52,53,54,55,56,57,58,59,60,61,62,63,64,65,66,67,68,69,70,71,72,73,74,75,76,77,78,79,80,81,82,83,84,85,86,87,88,89,90,91,92,93,94,95,96,97,98,99,100,101,102,103,104,105,106,107,108,109,110,111,112,113,114,115,116,117,118,119,120,121,122,123,124,125,126,127,128,129,130,131,132,133,134,135,136,137,138,139,140,141] from 762 studies (refer to Figure 1). The key characteristics of included studies are summarized in the Electronic Supplementary Table S2. Most studies were observational in design (n = 115), with 7 randomized controlled trials and 3 non-randomized trials also identified. Kidney transplant recipients constituted the largest population subgroup, featured in 95 cohorts, followed by liver (n = 39), heart (n = 29), and lung (n = 25) transplant recipients, with several studies (n = 38) including multi-organ transplant populations. The age of participants across studies ranged from 13.5 to 84 years, and males consistently represented the predominant proportion of the study populations, accounting for over 60% of participants. The most frequently administered vaccines were mRNA-based (n = 123), specifically the BNT162b2 (Pfizer-BioNTech) (n = 111) and mRNA-1273 (Moderna) platforms (n = 62), evaluated across various dosing schedules from one to five doses, with two- (n = 87) and three-dose (n = 30) regimens being the most common. One study administered double dose mRNA-1273 vaccine. The timing of seroconversion assessment post-vaccination varied widely, ranging from 10 days to over 12 months, though most studies (n = 103). Risk of bias assessment revealed that majority of the included studies had low risk amongst RCTs and moderate quality amongst the observational designs.

### 3.2. Seroconversion with COVID-19 Vaccines

Details of the seroconversion reported in each included study are summarized in the Electronic Supplementary Table S3. The overall pooled seroconversion proportion was estimated to be 0.49 (95% CI, 0.43 to 0.55), reflecting 12,136 events. To view the comprehensive forest plot representing the individual data points for all 125 included studies, please refer to Electronic Supplementary Figure S1. However, a significant degree of heterogeneity was observed across the studies, as evidenced by the high I^2^ statistic of 94.2% (*p* < 0.001) due to which the overall pooled estimate should be interpreted with caution as a aggregate of diverse clinical contexts rather than a definitive predictor for individual patients.

### 3.3. Seroconversion According to Vaccine Types

**Figure 2:**
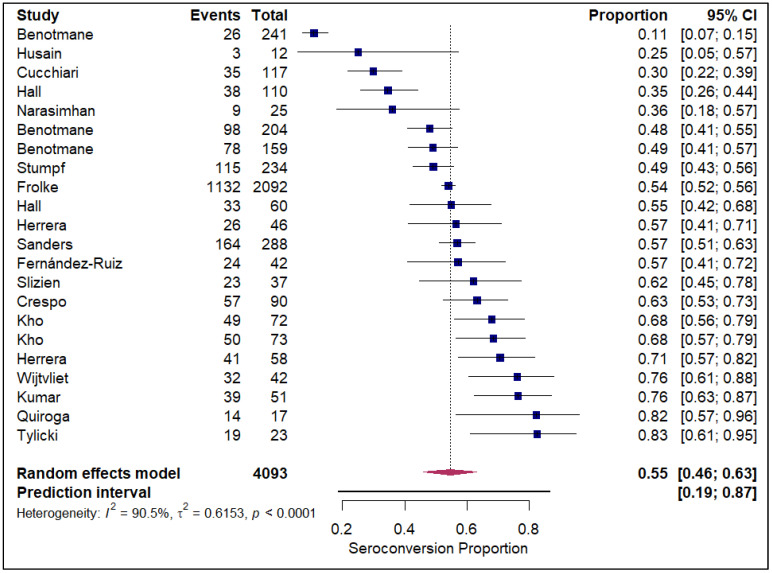
Forest plot for seroconversion according to mRNA-1273 vaccine. The figure displays meta-analysis of seroconversion proportions for the mRNA-1273 vaccine. The squares represent individual study proportions, the horizontal lines represent the 95% confidence intervals (CI), and the diamond at the bottom of each plot represents the overall pooled estimate.

**Figure 3:**
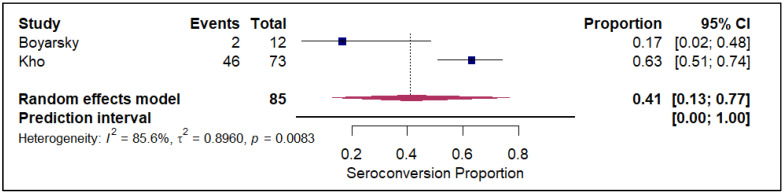
Forest plot for seroconversion according to Ad26.COV2.S vaccine. The figure displays meta-analysis of seroconversion proportions for the Ad26.COV2.S vaccine. The squares represent individual study proportions, the horizontal lines represent the 95% confidence intervals (CI), and the diamond at the bottom of each plot represents the overall pooled estimate.

**Figure 4:**
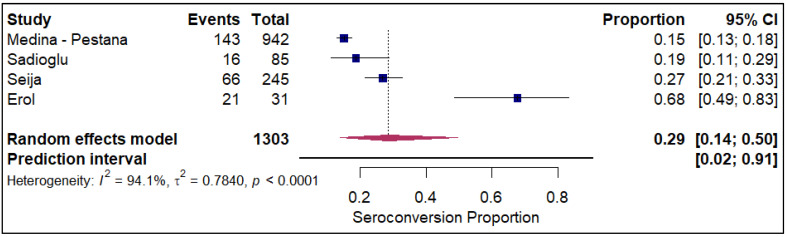
Forest plot for seroconversion according to CoronaVac vaccine. The figure displays meta-analysis of seroconversion proportions for the CoronaVac vaccine. The squares represent individual study proportions, the horizontal lines represent the 95% confidence intervals (CI), and the diamond at the bottom of each plot represents the overall pooled estimate.

The meta-analysis of seroconversion proportions stratified by vaccine type reveals distinct differences in pooled estimates and levels of heterogeneity across the four assessed vaccines. The BNT162b2 vaccine (refer to Electronic Supplementary Figure S2) demonstrated an overall pooled seroconversion proportion at 0.49 (95% CI: 0.41 to 0.57) across 7213 events, though it was associated with high heterogeneity (I^2^ = 93.3%). The mRNA-1273 vaccine (refer to Figure 2) showed a high pooled proportion of 0.55 (95% CI, 0.46 to 0.63) based on 4093 events, also exhibiting significant heterogeneity (I^2^ = 90.5%). In contrast, the Ad26.COV2.S vaccine (refer to Figure 3), analyzed with fewer events (n = 85), had a lower pooled seroconversion proportion of 0.41 (95% CI, 0.13 to 0.77), and similarly high heterogeneity (I^2^ = 85.6%). The Coronavac vaccine (refer to Figure 4) displayed the lowest pooled seroconversion proportion at 0.29 (95% CI, 0.14 to 0.50) from 1303 events, while still being highly heterogeneous (I^2^ = 94.1%).

### 3.4. Seroconversion According to Type of Organ Transplant

**Figure 5:**
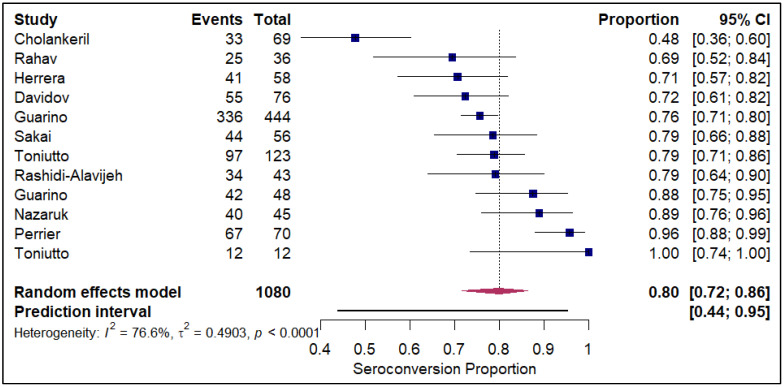
Forest plot for seroconversion in Liver transplant recipients. The figure displays meta-analysis of seroconversion proportions for liver transplant recipients. The squares represent individual study proportions, the horizontal lines represent the 95% confidence intervals (CI), and the diamond at the bottom of each plot represents the overall pooled estimate.

**Figure 6:**
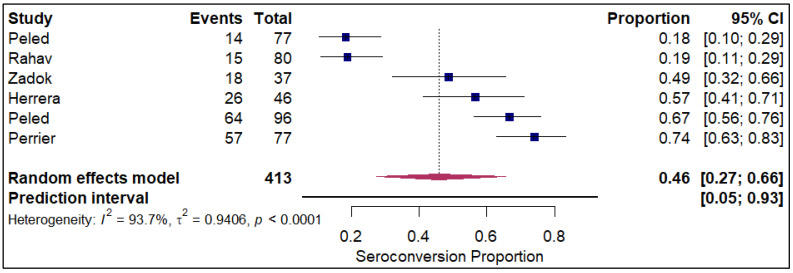
Forest plot for seroconversion in Heart transplant recipients. The figure displays meta-analysis of seroconversion proportions for heart transplant recipients. The squares represent individual study proportions, the horizontal lines represent the 95% confidence intervals (CI), and the diamond at the bottom of each plot represents the overall pooled estimate.

**Figure 7:**
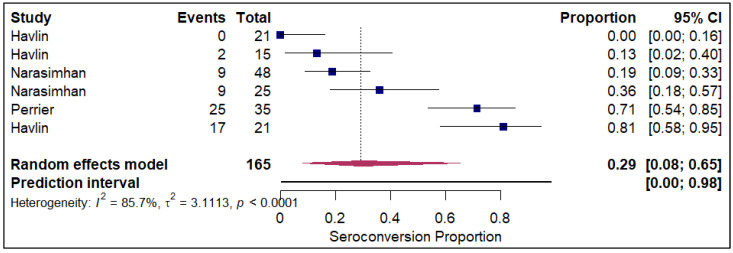
Forest plot for seroconversion in Lung transplant recipients. The figure displays meta-analysis of seroconversion proportions for lung transplant recipients. The squares represent individual study proportions, the horizontal lines represent the 95% confidence intervals (CI), and the diamond at the bottom of each plot represents the overall pooled estimate.

**Figure 8:**
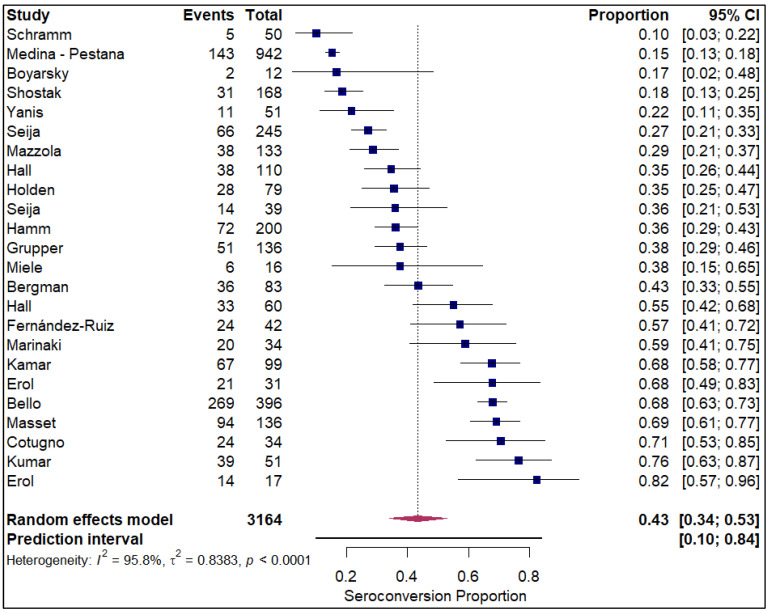
Forest plot for seroconversion in multiple organ transplant recipients. The figure displays meta-analysis of seroconversion proportions for multiple organ transplant recipients. The squares represent individual study proportions, the horizontal lines represent the 95% confidence intervals (CI), and the diamond at the bottom of each plot represents the overall pooled estimate.

The forest plots of pooled seroconversion proportions among solid organ transplant recipients revealed considerable differences in immune response across different organ groups. The pooled seroconversion proportions generally followed a decreasing trend, with the highest rates observed in liver transplant recipients (0.8; 95% CI: 0.72, 0.86; refer to Figure 5), followed closely by kidney transplant recipients (0.47; 95% CI: 0.39, 0.54; refer to Electronic Supplementary Figure S3) and heart transplants (0.46; 95% CI: 0.27, 0.66; refer to Figure 6). Conversely, the immune response was markedly blunted in lung transplant recipients (0.29; 95% CI: 0.08, 0.65; refer to Figure 7) and multiple organ transplant recipients (0.43; 95% CI: 0.34, 0.53; refer to Figure 8).

### 3.5. Seroconversion According to Prior COVID-19 Infection Status

**Figure 9:**
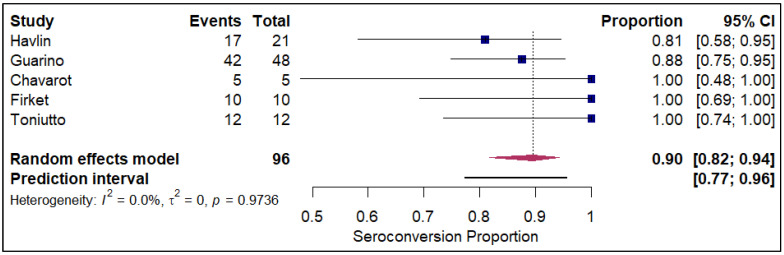
Forest plot for seroconversion amongst those with history of prior COVID-19 infection. The figure displays meta-analysis of seroconversion proportions for those with prior history of COVID-19 infection. The squares represent individual study proportions, the horizontal lines represent the 95% confidence intervals (CI), and the diamond at the bottom of each plot represents the overall pooled estimate.

The analysis of seroconversion proportions stratified by prior SARS-CoV-2 infection status revealed a substantial difference in immune response following vaccination. In the cohort of patients with a prior history of COVID-19 infection (refer to Figure 9), the pooled seroconversion proportion was exceptionally high at 0.90 (95% CI, 0.82 to 0.94) across 96 events. This strong and consistent response is further supported by the minimal heterogeneity observed in this group (I^2^ = 0%), suggesting a highly predictable serological response to vaccination among those with pre-existing natural immunity. Conversely, in the large cohort without a prior history of infection (refer to Electronic Supplementary Figure S4), the pooled seroconversion proportion was significantly lower at 0.49 (95% CI, 0.46 to 0.52) across 12,703 events. This group exhibited an extremely high degree of heterogeneity (I^2^ = 94.7%). Strikingly, stratification by prior SARS-CoV-2 infection was the only analysis that successfully resolved study-level heterogeneity. In the prior-infection cohort, the I^2^ was reduced to 0%, suggesting that natural infection acts as a powerful unifying immunologic stimulus that overrides the variability seen in infection-naïve populations. In contrast, the infection-naïve group retained extreme heterogeneity (I^2^ = 94.7%), indicating that other factors (e.g., immunosuppressive intensity) continue to drive variance in this subgroup.

### 3.6. Seroconversion According to Number of Dosage Regimens

**Figure 10:**
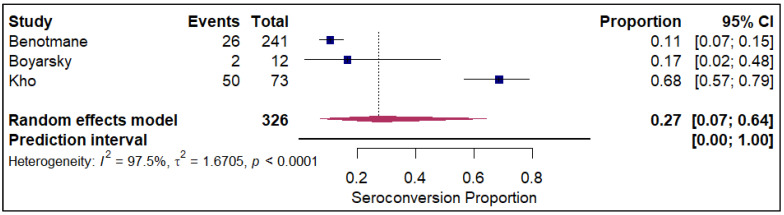
Forest plot for seroconversion with one dose of vaccine. The figure displays meta-analysis of seroconversion proportions for those with one dose of vaccine. The squares represent individual study proportions, the horizontal lines represent the 95% confidence intervals (CI), and the diamond at the bottom of each plot represents the overall pooled estimate.

**Figure 11:**
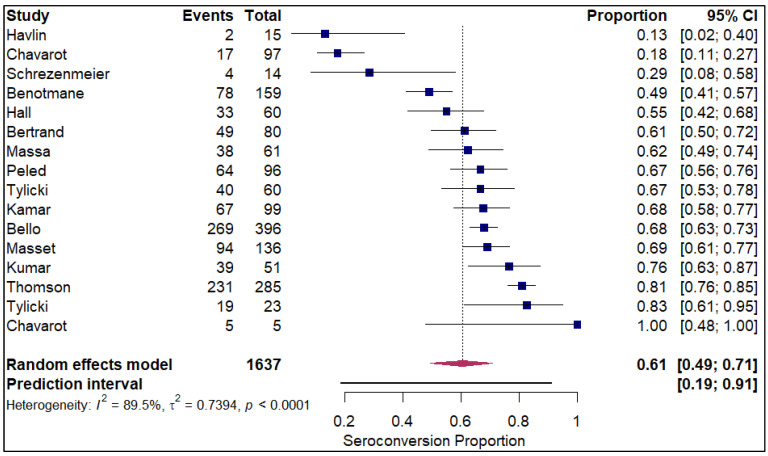
Forest plot for seroconversion with three doses of vaccine. The figure displays meta-analysis of seroconversion proportions for those with three doses of vaccine. The squares represent individual study proportions, the horizontal lines represent the 95% confidence intervals (CI), and the diamond at the bottom of each plot represents the overall pooled estimate.

**Figure 12:**
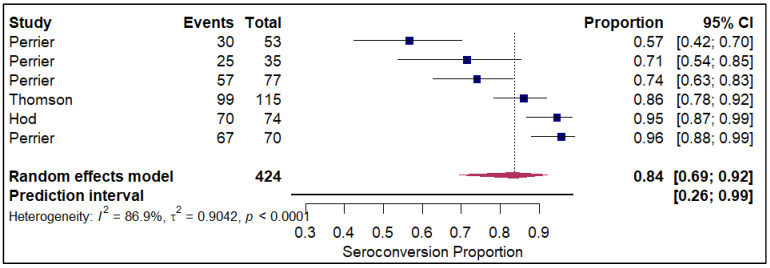
Forest plot for seroconversion with four doses of vaccine. The figure displays meta-analysis of seroconversion proportions for those with four doses of vaccine. The squares represent individual study proportions, the horizontal lines represent the 95% confidence intervals (CI), and the diamond at the bottom of each plot represents the overall pooled estimate.

The analysis of seroconversion proportion as a function of the number of vaccine doses administered reveals a clear, positive dose–response relationship. The seroconversion proportion exhibited a progressive increase with each additional dose, commencing at 27% after a single dose (refer to Figure 10). This proportion nearly doubled to 45% after the second dose (refer to Electronic Supplementary Figure S5). A further significant gain was observed with the third dose, which raised the seroconversion proportion to 61% (refer to Figure 11). The highest proportion was achieved after the fourth dose, reaching 84% (refer to Figure 12).

### 3.7. Bootstrap Analyses

The bootstrap analyses confirmed higher seroconversion rates observe with mRNA-1273 vaccine type, liver transplants, prior COVID-19 infections and higher number of vaccine doses (refer to Table 1).

### 3.8. Leave-One-out Sensitivity Analyses and Publication Bias Analyses

Leave-one-out sensitivity analysis revealed that no significant impact was observed on the pooled estimates with the removal of data from each study included in most of the analyses except for CoronaVac vaccine, lung and heart transplant recipients, one dose and those with prior COVID-19 infection (refer to Electronic Supplementary Table S4).

Egger’s regression analyses did not reveal presence of any publication bias (refer to Table 2).

### 3.9. Seroconversion According to Definitions

When stratified by antibody specificity, anti-RBD antibodies demonstrated the highest seroconversion rate at 54.6% (95% CI: 48.1–61.0%), while anti-spike IgG antibodies showed a slightly lower rate of 48.7% (95% CI: 42.8–54.7%). Notably, the combination of both anti-spike IgG and anti-RBD antibodies yielded the lowest seroconversion proportion at 27.1% (95% CI: 15.4–43.2%). Quantitatively, our results indicate that the use of anti-RBD assays alone may overestimate vaccine-induced immunity by approximately 27% relative to dual-antibody testing. Conversely, the requirement for dual anti-spike and anti-RBD positivity introduces a conservative bias that likely underestimates biological priming. These findings suggest that the choice of assay and the stringency of cutoff thresholds can shift pooled seroconversion estimates by nearly 27% in either direction, contributing significantly to the observed inter-study heterogeneity.

### 3.10. Safety Profile of COVID-19 Vaccines in Organ Transplants

Of the 125 included studies, safety profile has been reported only in 36. Details of safety concerns reported in the studies included are mentioned in the Electronic Supplementary Table S5. In general, the COVID-19 vaccines demonstrated a favorable safety profile. Local and systemic adverse events were predominantly mild to moderate in severity, with the mostly local reaction being injection-site pain (median report rate: 59%) and frequent systemic events including fatigue (median rate: 32%), headache (median rate: 22%), and myalgia (median rate: 19.5%). Serious adverse events and episodes of acute allograft rejection were infrequent, with only isolated cases reported, such as biopsy-proven acute rejection [36,42,56,73,126,133]. A substantial number of studies explicitly concluded that the vaccines were safe, with no vaccine-related deaths or significant safety signals, and several noted no observed episodes of acute organ rejection. However, the absence of safety signals reflects limited reporting rather than confirmed safety equivalence.

## 4. Discussion

### 4.1. Key Findings

This systematic review and meta-analysis of COVID-19 vaccination in solid organ transplant recipients establishes that this vulnerable population exhibits a significantly blunted seroconversion rate compared to the public, with an overall pooled proportion of only 0.49 (95% CI, 0.43 to 0.55) across 125 studies, underscoring the challenge of achieving protective immunity in this highly immunocompromised group. Crucially, the analysis identified several powerful determinants of vaccine success: prior SARS-CoV-2 infection was the strongest predictor of a robust response, resulting in an exceptionally high seroconversion rate of 0.90, effectively normalizing the immune response in SOTRs with hybrid immunity. Furthermore, seroconversion rates demonstrated a clear, positive dose–response relationship, escalating progressively from 27% after one dose to 84% after four doses, highlighting the critical necessity of booster regimens. While the immune response varied significantly across vaccine types and transplanted organs, with Liver recipients and mRNA-1273 vaccines showing relatively higher proportions than Lung recipients and CoronaVac, the consistent finding of high heterogeneity across nearly all subgroups emphasizes that vaccination strategies must be highly individualized, considering both the patient’s history and the specific organ and regimen.

### 4.2. Comparison with Existing Literature

Our findings not only align with the results of previous syntheses but also substantially advance the understanding of COVID-19 vaccine effectiveness in SOTRs by providing a single, comprehensive quantitative framework assessing organ type, dose number, and prior infection status. Earlier meta-analyses consistently reported pooled seroconversion rates across different dosing regimens ranging from 20% to 60% [6,7,8,9,12]. Our overall pooled seroconversion rate of 0.49 (95% CI: 0.43–0.55) closely mirrors these findings, validating the consistency of the attenuated immune response in SOTRs while offering finer granularity by dissecting outcomes by transplant type, vaccine platform, and dose sequence. Our study presents robust organ-specific gradients that reaffirm and provide more precise estimates than prior syntheses. Earlier pooled analyses identified liver transplant recipients as the most responsive subgroup and lung transplant recipients as the least responsive, reflecting fundamental differences in immunosuppressive intensity and graft immunobiology. For instance, Chen et al. reported second-dose seroconversion rates of approximately 64.5% in liver, 37.6% in kidney, 50.3% in heart, and 28.4% in lung transplant recipients [12]. Our pooled estimates confirm this established hierarchy but reveal a greater separation among groups. Liver transplant recipients demonstrated the highest seroconversion rate (0.80; 95% CI: 0.72–0.86), while kidney and heart recipients exhibited intermediate rates (0.47; 95% CI: 0.39–0.54 and 0.46; 95% CI: 0.27–0.66, respectively). Lung transplant recipients remained the least responsive, with a pooled rate of 0.29 (95% CI: 0.08–0.65). These patterns align with the biological plausibility that liver transplantation is often associated with a more tolerogenic immune environment, permitting reduced immunosuppression. In contrast, lung transplantation necessitates more intensive regimens due to a higher risk of rejection and persistent immune stimulation, factors that profoundly impair B-cell regulation and antibody production [14,15]. Our platform-specific analysis facilitates a robust assessment of relative seroconversion rates. Earlier meta-analyses [7,8,11] indicated that mRNA-1273 demonstrates superior seroconversion proportions compared with BNT162b2 after two or three doses. For instance, Manothummetha et al. reported seroconversion rates of 44% (0–79.1%) for BNT162b2 and 51.4% (29.9–76.2%) for mRNA-1273 [8,9]. Similarly, our analysis showed that mRNA-1273 achieved the highest pooled seroconversion rate (0.55; 95% CI: 0.46–0.63), followed by BNT162b2 (0.49; 95% CI: 0.41–0.57). The Ad26.COV2.S vaccine (0.41; 95% CI: 0.13–0.77) and CoronaVac (0.29; 95% CI: 0.14–0.50) consistently elicited lower responses. Several factors may contribute to this difference: mRNA-1273 delivers a higher mRNA dose and utilizes a longer dosing interval (four weeks versus three weeks), which is consistent with evidence that extended intervals improve antibody responses. Additionally, differences in lipid nanoparticle formulations (SM-102 in mRNA-1273 versus ALC-0315 in BNT162b2) may also play a role [11]. A major advance of this study is comprehensive stratification by prior SARS-CoV-2 infection status. Most previous analyses excluded these patients, limiting interpretation to infection-naïve cohorts. In contrast, our analysis demonstrates that prior infection significantly enhances vaccine-induced immunity, with pooled seroconversion increasing from 0.49 (95% CI: 0.46–0.52) in infection-naïve recipients to a robust 0.90 (95% CI: 0.82–0.94) in those with documented prior COVID-19. This suggests that the immune priming achieved through prior infection induces memory B and T cells more effectively than vaccination alone in immunosuppressed populations [16].

### 4.3. Dose–Response and Assay Variability

The dose-stratified findings reinforce the reliability of additive immunologic gains. Previous syntheses reported increases from 9.5% after the first dose to 55.1% after the third [5,12]. Our results show similar stepwise increases: from 27% after one dose to 45% after two, 61% after three, and 84% after four doses. This progressive increase supports a model where repeated antigen exposure incrementally improves responsiveness, even under ongoing immunosuppression. These findings underscore the urgent need for repeated vaccination to achieve protective antibody levels, including the consideration of fifth or subsequent doses for non-responders [6,7,8,9,10,11,12,13]. We also identified that the choice of serological assay exerts a profound quantitative influence on pooled estimates. Seroconversion proportions fluctuated by nearly 27% depending on the target: anti-RBD assays were the most sensitive (54.6%), while requiring both anti-spike and anti-RBD positivity yielded the lowest proportion (27.1%). These discrepancies are likely driven by assay sensitivity and manufacturer-defined cutoffs optimized for the general population. This highlights the urgent need for standardized serological protocols to ensure that meta-analytic estimates accurately reflect the immunologic status of SOTRs. Considering the heterogeneity observed, we suggest not overinterpreting the pooled point estimates as these values represent aggregate measures amidst significant clinical and methodological variability.

### 4.4. Comparison with Immunocompetent Populations and Safety

Our findings stand in sharp contrast to the high-immunogenicity benchmark in immunocompetent adults, where pooled seroconversion rates reach 92.64% [142], with mRNA vaccines achieving near-universal (99.5%) neutralizing antibody generation [143]. Regarding safety, the theoretical concern that vaccination might trigger acute allograft rejection appears unsupported by current data. Reported cases of biopsy-proven rejection were rare and manageable with standard adjustments. This mirrors the safety profile of other essential immunizations, such as influenza and pneumococcal vaccines, where the risk-benefit ratio heavily favors vaccination. The risk of severe disease or death following natural infection in SOTRs is markedly higher than any potential risks associated with the vaccine.

The major strength of this meta-analysis lies in its comprehensive scope, encompassing 125 studies and providing the most robust quantitative evidence to date through novel stratifications by organ type, dose number (up to four), and, critically, prior SARS-CoV-2 infection status, which clarified the powerful effect of hybrid immunity. However, the study is subject to several limitations. Foremost among these is the pervasive, high level of heterogeneity across most subgroups, stemming from differences in immunosuppressive regimens, specific antibody assays and their defined thresholds, follow-up times, and patient comorbidities, which restricts the generalizability of the overall pooled estimates. Furthermore, most of the included articles were observational, limiting causal inference. Although most studies have evaluated mRNA vaccines, evidence on other vaccine platforms remains limited. Substantial heterogeneity was observed in the assessment of seroconversion rates, as different assays were used and cutoff values defining seroconversion or a positive response varied across studies, potentially introducing measurement bias. In addition, safety outcomes were insufficiently reported. The persistent heterogeneity across most subgroups suggests that standard clinical variables such as organ type or vaccine platform only modestly account for the variance. The reduction of I^2^ to 0% in the prior-infection subgroup identifies hybrid immunity as a major primary driver of immunologic consistency. Other sources of quantitative bias include the lack of standardized international units; assays with lower analytical cutoffs tend to overestimate clinical protection, while those using higher ‘protective’ thresholds underestimate the actual rate of biological seroconversion. Consequently, most studies were unable to establish a clear association between vaccination and serious adverse events or mortality, limiting robust conclusions regarding vaccine safety.

## 5. Conclusions

This comprehensive systematic review and meta-analysis definitively demonstrates that solid organ transplant recipients are profoundly hyporesponsive to COVID-19 vaccination, exhibiting a substantially diminished overall seroconversion rate compared to the general population. Our findings underscore that achieving protective immunity in this vulnerable group is highly dependent on an aggressive, multi-dose strategy, with seroconversion rates progressively increasing to over 84% after four doses, reinforcing the critical necessity of booster regimens. The most potent predictor of a successful response was prior SARS-CoV-2 infection, which effectively normalized seroconversion to 90%. Significant variability was identified across subgroups, with Liver recipients showing the highest response and Lung recipients the lowest, necessitating organ-specific clinical consideration. Given the generally favorable safety profile observed, we conclude that current evidence strongly supports the prioritization and sustained pursuit of at least three to four vaccine doses in SOTRs, coupled with careful monitoring, to maximize the likelihood of a protective immune response against SARS-CoV-2. However, the absence of safety signals reflects limited reporting rather than confirmed safety equivalence. Rare but clinically relevant outcomes (such as acute rejection) cannot be adequately assessed through the studies included. Furthermore, these results underscore the need for future vaccine trials in transplant populations to utilize organ-specific stratification and account for prior infection status as a critical covariate to mitigate baseline heterogeneity. Trial designs should transition from standard two-dose protocols toward longitudinal, multi-dose assessments that accurately capture the delayed and incremental immune kinetics characteristic of this highly immunosuppressed cohort.

## Figures and Tables

**Table 1:** Analysis of Pooled Seroconversion Proportions Derived from Bootstrap Resampling Analysis.

Category	Type	Bootstrap Mean of Seroconversion Proportion	Bootstrap 95% Lower CI of Seroconversion Proportion	Bootstrap 95% Upper CI of Seroconversion Proportion
Vaccine	BNT162b2	49.04	41.42	56.31
	Ad26.COV2.S	39.99	16.67	63.01
	CoronaVac	29.82	15.48	54.25
	mRNA-1273	54.73	45.85	61.76
Organ	Liver	79.5	72.39	87.23
	Kidney	46.64	38.93	53.61
	Mixed	42.99	33.74	51.93
	Lung	30.7	4.79	62.72
	Heart	45.96	27.32	65.59
Dose	1	30.37	10.79	68.49
	2	44.81	38.55	51.14
	3	60.81	48.58	69.52
	4	83.08	70.35	92.16
Prior COVID status	No	45.92	39.86	51.48
	Yes	91.99	85	100

**Table 2:** Egger’s regression analyses for publication bias assessment.

Category	Type	Egger Intercept	Egger SE	Egger t	*p*-Values
Dose	1 Doses	12.5076	3.3993	0.099	0.9374
	2 Doses	0.7641	0.1463	−0.674	0.5023
	3 Doses	1.6683	0.3647	−0.985	0.3413
	4 Doses	5.8416	1.1013	1.746	0.1558
Vaccine	mRNA-1273	1.0858	0.1651	0.128	0.8996
	BNT162b2	0.9252	0.2163	−1.082	0.2826
	CoronaVac	5.8498	0.4794	1.774	0.2181
Prior COVID infections	Yes	1.0339	0.3754	1.957	0.1453
	No	0.8197	0.1562	−0.7	0.4856
Organ transplantation	Kidney	−0.9718	0.1463	−1.207	0.2325
	Liver	1.1643	0.2974	1.259	0.2365
	Heart	11.099	3.0334	−1.288	0.2673
	Lung	3.4559	1.6706	−0.689	0.5287
	Mixed	2.6012	0.4221	1.246	0.2257

## Data Availability

Data is available with the corresponding author and shall be shared upon a reasonable request.

## Supplementary Materials

The following supporting information can be downloaded at: https://www.mdpi.com/article/10.3390/vaccines14010072/s1, Table S1: Search strategy; Table S2: Key characteristics of included studies; Table S3: Efficacy and immunogenicity of COVID-19 vaccines amongst the included studies; Table S4: Leave-one-out sensitivity analysis; Table S5: Safety of COVID-19 vaccines amongst the included studies; Figure S1: Forest plot for overall seroconversion; Figure S2: Forest plot for seroconversion with BNT162b2; Figure S3: Forest plot for seroconversion in Kidney transplant recipients; Figure S4: Forest plot for seroconversion amongst those without history of prior COVID-19 infection; Figure S5: Forest plot for seroconversion with two doses of vaccine.
