# Supplementary material for: Efficacy and Safety of mRNA-Based COVID-19 Vaccines in Solid Organ Transplant Recipients: A Systematic Review and Meta-Analysis
Source: Vaccines (Basel). 2026 Jan 8;14(1):72. doi: 10.3390/vaccines14010072 (PMC12846407; doi:10.3390/vaccines14010072)
Supplement: Supplementary file 1 [file vaccines-14-00072-s001.zip › vaccines-4076031-supplementary.pdf]

Electronic Supplementary Table 1. Search strategy.

(COVID-19 vaccine[Title/Abstract] OR COVID vaccine[Title/Abstract] OR Coronavirus vaccine[Title/Abstract] OR mRNA vaccine[Title/Abstract]) AND (random*[Title/Abstract] OR randomized[Title/Abstract] OR randomised[Title/Abstract]) AND (immun*[Title/Abstract] OR immunocompromised[Title/Abstract] OR immunosuppressed[Title/Abstract] OR steroids[Title/Abstract] OR chemotherapy[Title/Abstract] OR transplant[Title/Abstract] OR immunosuppressive[Title/Abstract] OR neoplasm[Title/Abstract] OR cancer[Title/Abstract] OR infection[Title/Abstract] OR autoimmune[Title/Abstract] OR malignancy[Title/Abstract] OR rheumatic[Title/Abstract] OR systemic lupus erythematosus[Title/Abstract] OR HSCT[Title/Abstract] OR CAR-T[Title/Abstract] OR transplantation[Title/Abstract] OR immunotherapy[Title/Abstract] OR tumors[Title/Abstract] OR leukemia[Title/Abstract] OR lymphoma[Title/Abstract] OR inflammatory[Title/Abstract] OR rheumatoid arthritis[Title/Abstract] OR psoriasis[Title/Abstract] OR myasthenia gravis[Title/Abstract] OR HIV[Title/Abstract] OR AIDS[Title/Abstract] OR connective tissue disorder[Title/Abstract] OR neutropenia[Title/Abstract] OR splenectomy[Title/Abstract] OR liver[Title/Abstract] OR kidney[Title/Abstract] OR corticosteroids[Title/Abstract] OR multiple sclerosis[Title/Abstract] OR sarcoidosis[Title/Abstract] OR neuromyelitis optica[Title/Abstract] OR transplantation[Title/Abstract])

Electronic Supplementary Table 2. Key characteristics of included studies.

Study (Author, Year); Country	Study Design	Population (N); median age, gender distribution	Organ transplantation Subgroup(s) & Key Details	Comorbid disorders	Vaccine(s) Administered (Doses)	Last follow-up Period (days) - Last day of follow up.
Azzi 2021, USA	Observational cohort	No prior Covid group: 76 patients, Median 60.5 years, 57% males.	Kidney, Median 4 years	Hypertension<br>Diabetes<br>Glomerular disease<br>Heart disease<br>Lung disease<br>Cancer	BNT162b2 or mRNA-1273 or Ad26.CoV.2. 2 doses for BNT162b2 and mRNA-1273, 1 dose for Ad26.CoV2. S.	Not reported
		Prior Covid group: 21 patients, 60.5 years, 57% males.				

Cholankeril, 2022, USA	Prospective Observational Study	69 patients, median 63 years. Male: Female: 48 / 21 (70% / 30%)	Liver, median 3.3 years	Obesity Diabetes Mellitus Chronic Kidney Disease stage III/IV.	BNT162b2. 2 doses.	The median time from the first vaccine dose to the antibody test was 81 days.
Westhoff 2021, Germany	Prospective interventional study – non randomized trial	10 patients, Mean age 59.5 years, 80% males	Kidney Transplant Recipients, duration not specified	Not reported	BNT162b2 x 2 + mRNA-1273. 3 doses	14 days after the third vaccination .

D'Offizi, 2021, Italy	Prospective Observational Cohort	61 patients, Median 59 years, 70% male.	Liver, 6 years	Diabetes Mellitus Obesity (BMI >30 Chronic Kidney Disease (eGFR <51 ml/min)	BNT162b2 or mRNA-1273. 2 doses.	2 weeks (approximately 14 days) after the second dose.
Sadioglu, 2021, Turkey	Cross-sectional prospective observational study	85 patients, Mean age 46.4 years; 47 Female / 38 Male (55.3% / 44.7%)	Kidney transplant recipients. Mean 82 months	Primary kidney diseases	Inactivated SARS-CoV-2 vaccine (CoronaVac; Sinovac Biotech), 2 doses	One month (median 31 days) after the second vaccine dose.

Sattler, 2021, Germany	Observational cohort study	39 patients, Mean age 57.38 years; 11 Female / 28 Male (28.21% / 71.79%)	Kidney transplant recipients, mean 8.15 years	Hypertension Diabetes Coronary heart disease History of malignancy	BNT162b2. 2 doses	Primary humoral/cellular analysis at Day 8 after booster (2nd dose)
Davidov, 2021, Israel	Prospective Observational Cohort	76 patients, median 64 years, 56.6% male	Liver, median 7 years	Hypertension Dyslipidaemia Diabetes Mellitus Chronic Kidney Disease	BNT162b2. 2 doses	Adverse events monitored for 30 days after vaccination. Serology tested at a mean of 38 days after the second dose.

Rashidi-Alavijeh, 2021, Germany	Prospective, single-center, observational cohort study	43 patients, median 57 years, 26 male (60.5%), 17 female (39.5%)	Liver, Median 8 years	Hepatocellular carcinoma Primary sclerosing cholangitis Alcohol-induced cirrhosis	BNT162b2. 2 doses	Antibody testing occurred a median of 15 days post–second dose.
Rincon-Arevalo 2021, Germany	Observational cohort study	40 patients, median 62.4 years, 70% male	Kidney, Median 5 years	Not quantified	BNT162b2. 2 doses	3 to 4 weeks after the second (booster) vaccination. Doses are 21 days apart

Balsby 2022, Denmark	Prospective case-control study	358 patients, median 57.2 years. 60.2% males	Kidney, Liver, Heart, Lung, combination . Duration not specified	Diabetes Mellitus<br>Cancer<br>Peripheral Vascular Disease<br>Chronic Pulmonary Disease<br>Heart Disease<br>Renal Disease<br>Cerebrovascular Disease<br>Rheumatic Disease<br>Liver Disease	BNT162b2 or mRNA-1273. 3 doses	Median time from third dose to blood sampling was 41.5 days
Bergman 2021, Sweden	Prospective open-label clinical trial (non-randomized).	83 SOT. Age median not reported. Male: 45 (51%) Female: 44 (49%)	Liver, kidney, kidney-pancreas. 6mo (n=33) with/ without MMF >6mowith MMF (n=20) >6mowithout MMF(n=36)	Not reported	BNT162b2. 2 doses	7 to 8 months.

Benotmane 2021 A, France	Observational cohort study	204 patients. Median 57.7 years. 130 males (63.8%)	Kidney, Median 6.2 years	Not reported	mRNA-1273. 2 doses	28-day post-vaccination point.
Marion, 2021, France	Observational, Retrospective cohort	Kidney patients: 217, Mean age: 59 years, 232 males (63%), 135 females (overall)	Kidney Liver Thoracic organs Pancreas. 108 months	Not reported	BNT162b2 or mRNA-1273. 2 doses	4 weeks (28 days) after the second dose.

		Liver patients: 58. Mean age: 59 years, 232 males (63%), 135 females (overall)				
		Thoracic organ patients: 33. Mean age: 59 years, 232 males (63%), 135 females (overall)				

		Pancreas patients: 5. Mean age: 59 years, 232 males (63%), 135 females (overall)				
Kamar 2021, France	Observational Cohort	101 patients. Median Age: Mean age 58 years. 69% male.	Kidney, Liver, Lung/Heart, Pancreas. 97 months.	Not reported	BNT162b2. 3 doses	4 weeks after the third dose.

Del Bello 2022, France	Retrospective observational study	396 patients, Mean age 59 years. 65% men	Solid organs, not specified, duration not reported	Not reported	BNT162b2. 3 doses	4 weeks after the third dose
Benotmane 2021 B, France	Single-center, prospective, observational cohort study.	242 patients, Median 57.7 yrs, 64.7% male.	Kidney, Median 6.4 years	Not reported	mRNA-1273. 1 dose	28 days after the first dose

Benotmane 2021 C. France	Observational Cohort	159 patients, Median Age: 57.6 years, 98 males / 61 females (61.6% / 38.4%)	Kidney, Median 5.3 years	Not reported	mRNA-1273. 3 doses	28 days after the 3rd dose
Cao 2022, USA	Retrospective observational study	37 patients, Median 64 years. 27 male (73%), 10 female.	Lung, Kidney, Heart, Liver, Heart–Lung, duration not reported	Not reported	mRNA. Pfizer or Moderna. 2 doses	Not reported

Cotugno 2022, Italy	Prospective observational study	34 patients. Age and gender not reported	Heart and Lung. Duration not reported	Not reported	BNT162b2. 2 doses	120 d after vaccination
Devresse 2021, Belgium	Prospective single-center cohort study	90 patients. median age: 60 years. 48% female	Kidney, Median 102 months	Not reported	BNT162b2 , 2 doses	1 month after the second vaccine dose.

Firket 2021, Belgium	Prospective cohort study	SARS-CoV-patient with prior infection: 10 patients. Median 52.7 years. 60% Female	Kidney, 77.8 months	Not reported	BNT162b2, 2 doses	15 days post-second dose (T3), with an additional testing point at 50 days for COVID-19-negative KTRs. .
		SARS-CoV-2 patients without prior infection: 10. Mean 49.7 years. 50% Female	Kidney, Mean 121.7 months			

Georgery 2021, Belgium.	Observational cohort study.	79 patients. Median 61, 48% male.	Kidney, 105 months	Not reported	BNT162b2, 2 doses.	28 days after a single dose
Masset 2021, France	Retrospective cohort study	456 patients, 60.3% male. Age not provided	Kidney and pancreas transplant recipients, mean 10.5 years	Diabetes Cardiovascula r disease Neoplasia history	BNT162b2. 3 doses	30 days after each vaccine dose

Griessbach 2022, Switzerland	Extension of a randomized controlled noninferiority trial (COVERALL-2)	26 patients, Age and gender not reported	Mixed SOT recipients, duration not specified	Not reported	mRNA-1273 or BNT162b2. 3 doses	8 weeks after receiving the third vaccination.
Havlin 2022, Czech Republic	Prospective cohort study	15 patients, Median 56.2 years,87% male (13/15), 13% female (2/15)	Lung. Median 1277 days	Not reported	BNT162b2. 3 doses	Immune responses: 3 weeks post–third dose; safety: 3 months post–third dose.

Hoffman 2021, The Netherlands	Observational cohort study	No COVID-19 before vaccination: 79 patients, age and sex not mentioned	Lung, Median 5.35 years	Not reported	BNT162b2 or mRNA-1273. 2 doses	6 weeks after the second vaccine dose.
		Had COVID-19 before vaccination: 12 patients, age and sex not mentioned		Not reported		

Holden 2021, Denmark	Observational cohort study.	80 patients median, 58.9 years, 55% male (44/80)	Kidney<br>Liver<br>Heart<br>Lung<br>Combined (Kidney/Liver, Kidney/Heart, Heart/Lung)	Not reported	BNT162b2. 2 doses	6 weeks after the second vaccine
Husain 2021. USA	Observational cohort study	Moderna group: 12 patients. Median 66 years, 11 female (39%), 17 male (61%)	Kidney, Median 8 years (overall)	Hypertension<br>Diabetes	mRNA. Moderna. 2 doses or Pfizer-BioNTech	Follow-up ranged from 12–59 days post–second dose.

		Pfizer: 16 patients, median 66 years, 11 female (39%), 17 male (61%)			Pfizer-BioNTech. 2 doses or mRNA Moderna	
Marinaki 2021, Greece	Observational cohort study	34 patients, ≤60 (17) >60 (17), Male 79.4% (27/34), Female 20.6% (7/34)	Kidney, Heart, median 11.1 years	Not reported	BNT162b2. 2 doses	10 days after the second vaccination

Massa 2021, France	Prospective monocentric longitudinal study – observational	61 patients, median 58.0 years, Male 72.1% (44/61), Female 27.9% (17/61)	Kidney, Median 4.5 years	Diabetes Obesity (BMI ≥30 kg/m²)	BNT162b2, 3 doses	28 days after the third vaccine
Chavarot 2021 A, France	Observational cohort study	101 patients, median age: 64 years, 68 men (67.3%)	Kidney, Median 59 months	Not reported	BNT162b2 . 2 doses	60 days after the vaccination

Chavarot 2021 B, France	Retrospective cohort study	COVID-19 naïve: 97 patients. Age and sex not reported.	Kidney, Median 47.5 months	Not reported	BNT162b2. 3 doses	44 days after the third dose
		Prior COVID-19: 5 patients. 66 years. 20% male.	Kidney, Median 19 months	Not reported	BNT162b2. 3 doses	44 days after the third dose

Noble 2021, France	Retrospective cohort study	57 patients, Mean 62 years, 18 (31.5%) females	Kidney, 136.5 months mean	Not reported	BNT162b2. 2 doses	1 month after the third vaccination
Pedersen 2021, Denmark	Observational cohort study	58 patients, Median 57.1 years (mean), 34 Female (58.6%), 24 Male (41.4%)	Kidney, Mean 6.8 years	Not reported	BNT162b2 . 2 doses	4 weeks after receiving a second dose

Medina-Pestana 2022, Brazil.	Single-center, prospective cohort, 12-month follow-up study.	3354 patients, Median 52 years, 60% male, 40% female.	Kidney, Simultaneous pancreas-kidney, median 7 years.	Diabetes mellitus	Inactivated SARS-CoV-2 vaccine. Sinovac Life Sciences. 2 doses	28 days after the first dose
Shostak 2021, Israel	Observational prospective cohort study	168 patients, Median 60.5 years, Female: 56 (33%); Male: 112 (67%)	Lung or heart-lung transplant, Categorized as <1 year (10%), 1-4 years (41%), 4-8 years (24%), >8 years (26%)	Not reported	BNT162b2. 2 doses	Follow up period was 68 days

Stumpf 2021 A, Germany.	Prospective single-center study	71 patients, Mean 57 years, 63% men	Kidney, Median 7.5 years	Glomerulonephritis<br><br>Hypertensive/ diabetic/vascular disease<br><br>Cystic kidney disease<br>Vasculitis	BNT162b2. 3 doses	16 weeks after initial dose
Stumpf 2021 B, Germany	Prospective, multicenter observational study	368 patients<br><br>(99 patients for BNT162b2),<br><br>Mean: 57.3 years, Male: 65.5% (241/368) (overall)<br>Female: 34.5% (127/368) (overall)	Kidney, Mean: 9.9 years (overall)	Cardiovascular disease<br><br>Diabetes mellitus<br><br>Lung disease<br>Cancer	BNT162b2. 2 doses or mRNA-1273	8 weeks after the first vaccination (the T2 time point).

		368 patients, (234 for mRNA-1273)<br><br>Mean: 57.3 years, Male: 65.5% (241/368) (overall)<br><br>Female: 34.5% (127/368) (overall)			mRNA-1273. 2 doses or BNT162b2	
Villanego 2021, Spain	Observational cohort study	91 patients. Median 59 years. 61 Male (67%), 36 Female (33%)	Kidney. Median 64 months	Not reported	mRNA-1273 / BNT162b2. 2 doses	Immunogenicity analysis was 1 month after second dose

Benning 2022, Germany.<br>.<br>.	Prospective observational cohort study	40 patients, median: 55 years, Female: 20/49 (41%) Male: 29/49 (59%) (overall)	Kidney, Median 8.1 years	Hypertension Diabetes Chronic artery disease Chronic lung disease Chronic liver disease Malignancy	3 doses of an mRNA vaccine BNT162b2 or mRNA 1273	5.9 months after the third vaccine dose
		7 patients, median: 55 years, Female: 20/49 (41%) Male: 29/49 (59%) (overall)			ChAdOx1 + 2 × mRNA. 3 doses	

		2 patients, median: 55 years,<br>Female: 20/49 (41%)<br>Male: 29/49 (59%)<br>(overall)			2 × ChAdOx1 + mRNA. 3 doses	
Bertrand 2021 A, France	Observational Cohort	45 patients, median 63.5 years, 23 males (51%), 22 females	Kidney, Median 6.9 years	Diabetes Mellitus<br>Hypertension	BNT162b2. 2 doses	1 month after the second dose.

Bertrand 2021 B, France	Observational Cohort	80 patients, Median Age: Mean 63.6 years. Male: Female Ratio: 48 / 32 (60% male).	Kidney, median 7.3 years	Not reported	BNT162b2. 3 doses	1 month after third dose
Bertrand 2021 C, France	Retrospective cohort study	235 patients, mean 60.8 years, 146 Male (62.1%), 89 Female (37.9%), 7.6 years	Kidney, 7.5 years (mean)	Not reported	mRNA BNT162b2. 2 doses	Follow-up after the second vaccine dose was 34.6 days.

Boyarsky 2021 A, United States	Prospective cohort study	Janssen vaccine group 12 participants. Age and gender not specified	Solid organ transplant recipients (specific types not listed)	Not reported	Ad26.COV 2.S. 1 dose or BNT162b2 or mRNA-1273/Moderna. 2 doses	28 days after the first dose.
		mRNA vaccine group: 725 participants. Age and gender not specified	Solid organ transplant recipients (specific types not listed)	Not reported	BNT162b2 or mRNA-1273. 2 doses or Replication incompetent adenovirus-type-26 vectored vaccine. 1 dose.	28 days after the first dose.

Boyarsky, 2021 B, USA	Prospective cohort study	436 patients, 55.9 years, Female: 61%, Male: 39%	Liver, Heart, Lung, Pancreas, Multiorgan and Kidney.	Not reported	BNT162b2 or mRNA-1273. 1 dose	20 days, after the first vaccine dose.
Boyarsky, 2021 C USA	Prospective cohort study	658 patients, age not mentioned 380 F / 268 M (40%)	Liver, Heart, Lung, Pancreas, Multiorgan and Kidney.	Not reported	BNT162b2 or mRNA-1273. 2 doses	29 days after the second vaccine dose.

Brandstetter 2022, Austria	Retrospective cohort study	324 patients, Median 60.6 years. 66.9% male, 33.0% female.	Kidney, Median 7.0 years	Diabetes Coronary artery disease Congestive heart failure Peripheral occlusive vascular disease Cerebrovascu lar disease	mRNA-1273 and BNT162b2. 4 doses	4-5 weeks after the last 4th dose
Bruminhent 2022, Thailand	Randomized controlled trial (non-blinded)	mRNA vaccine: 40 patients, median 50 years, 62% male (overall)	Kidney,46 months (overall)	Not reported	doses of CoronaVac + 1 ChAdOx1 dose + BNT62b2 or mRNA 1273 or 2 doses of ChAdOx1 + BNT62b2 or mRNA 1273	2 weeks after additional vaccine dose

		Viral vector vaccine group (V group): 37 patients median 50 years, 62% male (overall)	Kidney,46 months (overall)	Not reported	2 doses of CoronaVac + 1 ChAdOx1 dose + ChAdOx1 nCoV-19 or 2 doses of ChAdOx1 + ChAdOx1 nCoV-19	2 weeks after additional vaccine dose
Buchwinkler 2021, Austria	Retrospective cohort study	216 patients, 59.9 years, 32% female.	Kidney, duration not given	Cardiovascular disease<br><br>Cerebrovascular disease<br><br>Diabetes mellitus<br><br>Active/former malignancy	BNT162b2 or mRNA-1273. 2 doses	91 days after the first vaccine dose.

Chukwu 2022, United Kingdom	Retrospective observational cohort study	373 patients, Mean 55 years, 60% male (228/373)<br><br>BNT162b2 (Pfizer–BioNTech): 216 patients	Kidney, Median 91 months.	Diabetes Cardiovascular disease Post-transplant cancer (excluding non-melanoma skin cancer)	BNT162b2. 2 doses or AZD1222	155 days after the second dose
		373 patients, Mean 55 years, 60% male (228/373) AZD1222 (Oxford-AstraZeneca): 84 patients			AZD1222. 2 doses or BNT162b2	

Crane 2021, USA	Observational cohort study	25 patients, median 19 years old, 11 (44%) female, 14 (56%) male	Kidney, Median 5 years.	Not reported	BNT162b2 or mRNA-1273. 2 doses	median of 45 days.
Crespo 2021, Spain	Observational prospective cohort study	90 patients, Mean 59.7 years (mean), 35 females (38.9%), 55 males (61.1%)	Kidney, 26 patients were transplanted <1 year ago, and 64 patients were transplanted >1 year ago.	Arterial Hypertension Diabetes Mellitus Cardiovascula r Disease Pulmonary Disease	mRNA-1273. 2 doses	28 days after the second vaccine dose.

Cucchiari 2021, Spain	Observational cohort study	148 patients, Mean 57.62 years, 70.9% male	Kidney, Kidney-Pancreas. Median 1.65 years.	Diabetes Lymphopenia (<1000/mm^3)	mRNA-1273.2 doses	2 weeks after the second vaccine dose.
Danthu 2021, France	Prospective observational study.	74 patients, 64.8 years, 38.9% female.	Kidney, median 6.42 years.	Diabetes Ischemic heart disease	BNT162b2. 2 doses	Day 58 after the first injection.

Dov 2022, Israel	Prospective cohort study	252 patients, Mean 54 years, 33% female.	Kidney, median 4 years.	Hypertension Diabetes Cancer	BNT162b2. 2 doses	5 months and 19 days
Fernández-Ruiz 2021, Spain	Observational cohort study	44 patients, Mean 52.4 years. 61.4% male.	Kidney, liver, kidney-pancreas, liver-kidney. median 2.3 years	Hypertension Diabetes mellitus Dyslipidemia Cardiovascular disease Chronic pulmonary disease	mRNA-1273. 2 doses	2 months after the first dose

Frolke 2022, The Netherlands.	Prospective observational cohort study	2092 patients, 57.05 years, 58.37% male.	Kidney, 7.5 years	Hypertension<br>Diabetes<br>Cardiovascular Disease<br>Chronic Lung Disease<br>Heart Failure<br>Stroke<br>Peripheral Vascular Disease<br>Past Malignancy<br>Coronary Disease<br>Autoimmune Disease	mRNA-1273. 2 doses or BNT162b2 and mRNA-1273 or ChAdOx1-nCov19/AstraZeneca or Ad26.CoV2.S/J&J, undefined.3rd dose	8 weeks (56 days) after a third vaccination

		1401, 57.8 years (SD 11.6), 788 (56.2%) males	Kidney. 8.2 years	Hypertension<br>Diabetes<br>Cardiovascular disease<br>Chronic Lung Disease<br>Heart Failure<br>Stroke<br>Peripheral Vascular Disease<br>Liver Cirrhosis	mRNA-1273. 2 doses or BNT162b2 and mRNA-1273 or ChAdOx1-nCov19/AstraZeneca or Ad26.CoV2.S/J&J, undefined.3rd dose	8 weeks (56 days) after a third vaccination
Grupper 2021, Israel	Observational cohort study	136 patients, Mean 58.6 years. 18.3% female	Kidney, kidney–pancreas, kidney-after-liver, kidney–liver.	Hypertension<br>Diabetes mellitus<br>Ischemic heart disease<br>BMI >30	BNT162b2. 2 doses	10 to 20 days after the second vaccine dose.

Guarino 2022, Italy	Prospective Cohort Study	No Prior COVID): 444 patients, 65.27 years, 74.8% males	Liver, 10 to 15 years	Diabetes Cardiovascular disease Chronic kidney disease Respiratory disease Active cancer Previous cancer<br><br>.	BNT162b2 mRNA. 2 doses	88 days after the second dose.
		Previous COVID-19: 48 patients. 63.51 years. 39 (81.25%) males.	Liver, 13.96 years			

Hall 2021 A, Canada	Prospective observational cohort study.	127 patients. Median 66.2 years. 69.3% male.	Kidney: 30<br>Liver: 15<br>Lung: 33<br>Heart: 18<br>Kidney-pancreas: 28<br>Other: 3<br>Median 2.96 years.	Not reported	mRNA-1273. 2 doses	at least 6 weeks after the second vaccine dose.
Hall 2021 B, Canada	Double-blind, Randomized, Placebo-Controlled Trial	60 patients, median 66.6 years, gender not specified	SOT (not specified)	Not reported	mRNA-1273. 3 doses	Primary outcome was measured at Month 4 (approxi mately 60 days after the third dose/interv ention)

Hallet 2021, USA	Observational cohort study	Heart transplant recipient:134 patients. 18–39: 9 (31%), 40–59: 20 (53%), ≥60: 34 (52%). Male: 34 (52%), Female: 30 (45%)	Heart, <3 years: 10 (26%), 3–6 years: 19 (46%), 7–11 years: 13 (65%), ≥12 years: 22 (63%)	Not reported	mRNA-1273 and BNT162b2. 4 doses	Antibody samples were collected at a median of 29 days after the second dose.
		Lung transplant recipients: 103 patients. 18–39: 2 (12%), 40–59: 11 (41%), ≥60: 15 (25%) Male: 14 (34%), Female: 13 (22%)	Lung, <3 years: 10 (28%), 3–6 years: 7 (21%), 7–11 years: 5 (28%), ≥12 years: 6 (37%)			

Hamm 2022, Denmark	Prospective cohort study	200 patients and median 57 years, 55% male.	Liver<br>Kidney<br>Lung including 2 heart-lung transplants.<br>Median 5.8 years.	Cardiovascular disease<br>Chronic pulmonary disease<br>Diabetes mellitus<br>De novo non-skin cancer	BNT162b2. 2 doses	6 months after the first vaccine dose.
Harberts 2022, Germany	Prospective observational cohort study.	Patients: 106 Median 59 years, 39.6% female	Liver only. Median 8.8 years.	Hypertension<br>Diabetes<br>Chronic kidney disease (eGFR <45 mL/min)	BNT162b2 or mRNA-1273. 3 doses	A median of 29.5 days post-vaccination.

Haskin 2021, Israel	Prospective observational cohort study.	38 KTRs comprised the final study group. 18.6 ± 2.8 years (range 13.5–26.8). 25 Males (66%), 13 Females (34%).	Kidney only. Mean 7.3 years	Hypertension, lung disease, Immunodeficiency, Obesity.	BNT162b2. 2 doses	142 days from the first vaccine dose
Havlin 2021, Czech Republic	Prospective observational cohort study. .	LTRs patients' post-COVID: 33 patients	Lung. 1287 days.	Chronic obstructive pulmonary disease (COPD), Interstitial lung disease (ILD), Cystic fibrosis (CF)	BNT162b2. 2 doses	4–6 weeks after the second dose

		LTRs<br>Vaccinated patients: 48 patients. 52.1 years, 19 females.	Lung. 1552 days.	Chronic obstructive pulmonary disease (COPD): 10 (30.3%)<br><br>Interstitial lung disease (ILD): 15 (45.5%)<br><br>Cystic fibrosis (CF): 6 (18.2%)<br><br>Other: 2 (6.1%)	BNT162b2 . 2 doses	4–6 weeks after the second dose
Heinzel 2022, Austria	Randomized, single-blinded, controlled trial (RCT) / Secondary analysis of the RCT.	Homologous (mRNA) Group (n=85). Mean 61 years. 44% Female, 56% Male	Kidney. Median 4.8 years	Not reported	BNT162b2 or mRNA-1273. 3 doses or Viral Vector (Adenovirus) Ad26.COV2.S	60 and 120 days after the third vaccine dose.

		Heterologous (Vector) Group (n=84). Mean 61 years. 40% Female, 60% Male	Kidney. Median 4.9 years	Not reported	BNT162b2 or mRNA-1273 and Ad26.COV 2.S. 3 doses	60 and 120 days after the third vaccine dose.
Herrera 2021, Spain	Prospective observational cohort study.	Liver Transplant Recipients (LTR): 58 patients, Median 61.5 years. 31% female (18/58), 69% male	Liver. Median 4.6 years	Not reported	Moderna, mRNA-1273, 2 doses	4 weeks (28 days) after the second dose

		Heart Transplant Recipients (HTR): 46 patients, Median 60 years. 28% female (13/46), 72% male	Heart, median 6.3 years	Not reported	mRNA-1273, 2 doses	4 weeks (28 days) after the second dose
Hod A 2021, Israel	Prospective observational cohort study.	120 patients. Mean 59.7 years. 96 males (80%), 24 females (20%).	Kidney, Mean 5.8 years.	Hypertension Diabetes Ischemic Heart Disease Congestive Heart Failure	BNT162b2 2 doses	60 days after the second vaccine dose.

Kantauskaite 2021, Germany	Multicenter prospective observational study	225 patients. Median 62 years. 64.8% male	Kidney, 81 months	Not reported	BNT162b2 or mRNA-1273 . 2 doses	Mean 14 days after the second vaccine dose.
Karaba 2022, United States	Observational cohort study	25 patients. Median 59 years. Male: 52% (13/25), Female: 48% (12/25)	Kidney: Liver Liver-Kidney Pancreas Kidney-Pancreas Heart Lung. 4.3 years	Not reported	2 mRNA vaccine doses + mRNA or Ad26.COV2.S	Not reported

Kho 2022, The Netherlands.	Open-label, randomized clinical trial	Group 1A: Single Dose mRNA-1273 (Control Group), 73 patients randomized, 57.3 years, 25 (34%) Female, 48 (66%) Male	Kidney, 5.8 years	hypertension diabetes, coronary artery disease, heart failure, chronic lung disease, history of malignancy, auto-immune disease	Single dose mRNA-1273	Until 28 days after vaccine administrati on
		Group 1B: Double Dose mRNA-1273, 72 patients randomized, 58.5 years (SD 11.6), 27 (38%) Female, 45 (62%) Male	Kidney, 7.3 years		Double Dose mRNA-1273	Until 28 days after vaccine administrati on

		Group 1C: Heterologous Ad26.COV2-S, 73 randomized, 60.1 years (SD 12.4), 25 (34%) Female, 48 (66%) Male.	Kidney, 6.9 years		Heterologous Ad26.COV2-S. 1 dose	Until 28 days after vaccine administration
Korth 2021, Germany	Prospective cohort study	23 patients, Mean 57.7, 12 female (52%), 11 male (48%)	Kidney, Mean 11.4	Not reported	BNT162b2, 2 doses	Mean of 15.8 days after the second vaccine dose.

Kumar 2022, Canada	Prospective cohort study	60 patients, Median 66.9 years. Male: 37 (62%); Female: 23 (38%)	Kidney Liver, Lung, Heart, Pancreas/Kidney-Pancreas. Median 3.57 years	Not reported	mRNA-1273. 3 doses	The last day of follow-up for this study was 6 months after the first vaccine dose.
Marlet 2021, France.	Retrospective observational study.	After 3rd dose (n = 75): Age > 65 years: 26 patients (35%). Age < 50 years: 24 patients (32%). Female: 21 patients (28%)	Kidney, duration not specified	Not reported	BNT162b2 or mRNA-1273. 3 doses	95 days after the second dose.

Masset 2022, Germany	Observational cohort study.	Heterologous Vaccination Group: 28 patient, 59 years, 71.4% males	Kidney, 8.2 years	Not reported	ChAdOx1-nCov + mRNA booster. 3 doses or BNT162b2 or mRNA-1273	1-month post-dose 3
		mRNA-Exclusive Vaccination Group: 56 patients, Age mean 58.7 years 69.6% males	Kidney, 8.5 years	Not reported	BNT162b2 or mRNA-1273. 3 doses	1-month post-dose 3

Mazzola 2022, France.	Single-center retrospective cohort study.	143 patients, Median 61.0 years, Male: 71.3% (102/143)	Liver Kidney Heart Median 45.0 months.	Diabetes Cardiovascular complications	BNT162b2. 2 doses	28 days after the second dose.
Mrak 2022, Austria	Single-center, blinded, randomized controlled trial (RCT).	mRNA group: 24 patients, mean 63.4 years, mRNA group 41.7% female	Heart, Liver Kidney, Lung. Duration not specified	Not reported	BNT162b2 or mRNA-1273. 3 doses or BNT162b2 or mRNA-1273 (2 doses) and ChAdOx1 nCoV (1 dose)	4 weeks after the booster vaccination.

		Vector group: 22 patient, mean 61.2, 31.8% female.		Not reported	2 x BNT162b2 or mRNA-1273 and 1x ChAdOx1 nCoV or BNT162b2 or mRNA-1273. 3 doses	4 weeks after the booster vaccination .

Narasimhan 2021, USA	Observational cohort study	Pfizer-BioNTech Group: 48 patients. 20–39 years: (overall: 10/73) 40–59 (overall: 17/73) ≥60 years: (46/73). Male (overall): 47/73) Female: (overall): 26/73)	Lung. Median 40 months post-transplant	Not reported	BNT162b2 . 2 doses or mRNA-1273. 2 doses	Median 17.5 days after 2nd dose

		Moderna Group: 25 patients. 20–39 years: (overall: 10/73) 40–59 (overall: 17/73) ≥60 years: (46/73). Male (overall: 47/73) Female: (overall: 26/73)	Lung, Median 40 months post-transplant	Not reported	mRNA-1273. 2 doses or BNT162b2 . 2 doses	Median 19 days after 2nd dose
Osmanodja 2022, Germany	Retrospective cohort study	5th dose group, 40 patients. 63 years (56–72), 45% Female / 55% Male	Kidney, 7.2 years	Diabetes	BioNTech/ Pfizer's Comirnaty or Moderna's Spikevax. Up to 5 doses in total	One year after the vaccine

Ou 2021, USA	Prospective cohort study	609 patients, Median 58 years, 60% Female	Kidney , Median 7 years	Not reported	BNT162b2 or<br><br>mRNA-1273. 2 doses	Median of 29 days after the second vaccine dose.

Peled 2021 A, Israel	Prospective cohort study	77 heart recipients; Median age 62.0 years, 64% male	Heart; Median 7.4 years	HTN<br>Dyslipidemia<br>CKD<br>DM	BNT162b2. 2 doses	Mean 41 d after 2nd dose

Peled 2021 B, Israel	Prospective cohort study	96 heart recipients; Median age 61.0 years, 71% male, 29% female	Heart; Median 6.3 years	Hypertension<br><br>Diabetes Mellitus<br><br>Cardiac Allograft Vasculopathy	BNT162 b2. 3 doses	1 month after 3rd dose
Perrier 2022, France	Retrospective, observational, monocentric study	825 SOT recipients; Kidney group: 516 patients. Median age 61.2 years, 66.7% male, 33.3% female (overall)	Kidney, Liver, Heart Lung Median time since transplant 6.7 years	Not reported	BNT162b2. 4 doses	Study duration was 15 months.

		825 SOT recipients; Liver: 361 patients. Median age 61.2 years, 66.7% male, 33.3% female (overall)		Not reported	BNT162b2. 4 doses	
		825 SOT recipients; Heart: 108 patients. Median age 61.2 years, 66.7% male, 33.3% female (overall)		Not reported	BNT162b2. 4 doses	

		825 SOT recipients; Lung: 98 patients. Median age 61.2 years, 66.7% male, 33.3% female (overall)		Not reported	BNT162b2. 4 doses	
Quiroga 2022, Spain. .	Prospective, multicentric, observational study.	BNT162b2: 54 patients, overall age: 56 years, overall, 60% male	Kidney. Duration not specified	Diabetic Kidney Disease	BNT162b2, 2 doses or mRNA-1273 (Moderna), 2 doses	Day 28 after the final vaccine dose.

		mRNA-1273: 225 patients, Overall age: 56 years, overall 60% male	Kidney. Duration not specified		mRNA-1273. 2 doses or BNT162b2, 2 doses	Day 28 after the final vaccine dose.
Rabinowich 2021, Israel.	Observational cohort study.	80 patients, Mean 60.1 years, 30% female, 70% male	Kidney, Median 5 years	Hypertension<br>Hyperlipidemia<br>Diabetes<br><br>Chronic Kidney Disease (CKD) stage 3-5.	BNT162b2. 2 doses	10 weeks after the second dose

Rahav 2021, Israel	Prospective cohort study. .	Kidney Transplant: 111 patients, median age was 63.0 years (overall), 65.3% male (overall)	Kidney, Median 3.1 years	Not reported	BNT162b2. 2 doses	30 days following the second dose.
		Heart Transplant: 80 patients, median age was 63.0 years (overall), 65.3% male (overall)	Heart, Median 7.4 years	Not reported	BNT162b2. 2 doses	30 days following the second dose.

		Liver Transplant: 36 patients, median age was 63.0 years (overall), 65.3% male (overall)	Liver, Median 7.0 years	Not reported	BNT162b2. 2 doses	30 days following the second dose.
Ruether 2022, Germany	Prospective observational cohort study	138 patients. Mean 55.0 years, 59 (42.8%) female, 79 (57.2%) male. (overall, for LT group)	Liver, Median 7 years	Arterial Hypertension<br><br>Diabetes<br><br>Chronic Kidney Disease (eGFR 30-59 mL/min)	BNT162b2/ mRNA 1273 or AZD1222 / AZD1222 orAZD122 2 / mRNA Vaccinatio n. 2 doses	10 to 84 days after the second vaccination

Russo 2021, Italy	Retrospective analysis	82 Kidney Transplant Recipients; Median age: 58.5 yrs, 57.3% Male	Kidney; Median: 69 months	Hypertension Dyslipidemia Hyperuricemia Type 2 Diabetes.	BNT162b2 . 2 doses	Median of 43 days
Sanders 2022, The Netherlands	Prospective, Non-randomized controlled, multicenter study --	288 patients, Mean 56.1 years, 44.4% Female, 55.6% Male.	Kidney, Median 6.9 years	Hypertension<br><br>Diabetes mellitus<br><br>History of coronary artery disease<br><br>Heart failure<br><br>Chronic lung disease<br><br>History of malignancy<br><br>Autoimmune disease	mRNA-1273, 2 doses	Day 28 after the second vaccination .

Schmidt 2021, Germany.	Prospective observational study.	40 patients, Mean 54 years, 45% female, 55% male	Kidney Heart: Lung Liver Liver & Kidney Median 6.5 years	Not reported	ChAdOx1 nCoV-19 or BNT162b2 \ mRNA-1273. 2 doses	13–30 days after the first and the second vaccination .
Schramm 2021, Germany.	Prospective cohort study.	50 transplant recipients, Mean 54.5, 18 (45.0%)	Heart, Lung, Heart–Lung, Median 689 days.	Not reported	BNT162b2, 2 doses	21 days after the second vaccine dose

Schrezenmeier 2021, Germany.	Prospective cohort study. .	Heterologous ChAdOx1. Group: 11 patients ,59.7 years, 44.0% Female (overall)	Kidney, Mean 10.4 years	Hypertension Coronary heart disease Diabetes History of malignancy History of liver disease History of myocardial infarction	2 doses of BNT162b2 and 1 dose ChAdOx1, Vaxzevria, 3 doses or BNT162b2, 3 doses	Day 27 after the third vaccination .
		Homologous BNT162b2. Group: 14 patients, 59.7 (13.8) years (overall), 44.0% Female (overall)			BNT162b2, 3 doses or 2 doses of BNT162b2 and 1 dose ChAdOx1, Vaxzevria, 3 doses	

Schrezenmeier 2022, Germany.	Prospective interventional cohort study.	29 patients, mean 59.8. 8 years, 12 women (41.4%), 17 men (58.6%)	Kidney, 9.9 years.	Hypertension<br><br>Coronary Heart Disease<br><br>Diabetes<br><br>History of Malignancy Not reported	mRNA vaccines or vector-based vaccines + BNT162b2. 4 doses.	Day 32 after the fourth vaccination
Schwaighofer 2021, Austria.	Single-center, single-blinded, 1:1 randomized clinical trial.	Homologous mRNA Group: 99 patients, Mean 61.2 years, 42% Female, 58% Male.	Kidney, 4.68 years.	Not reported	mRNA vaccine primary + mRNA booster (Pfizer or Moderna). 3 doses or mRNA vaccine primary + viral vector (Ad26COVS1). 3 doses	4 weeks after 3rd vaccine

		Heterologous Vector Group: 98 patients, Mean 61.2 years, 41% Female, 59% Male	Kidney, 4.51 years	Not reported	mRNA vaccine primary + viral vector (Ad26COV S1). 3 doses or mRNA vaccine primary + mRNA booster (Pfizer or Moderna). 3 doses	
Seija 2022, Uruguay.	National multicentre cross-sectional study.	Inactivated Virus (CoronaVac): 245 patients Median 55 years (overall), 59% men (overall)	Kidney (95%)<br><br>Kidney-pancreas<br><br>(4.2%),<br><br>Median 61 months (overall)	Not reported	Inactivated Virus, Sinovac Biotech Ltd (CoronaVac), 2 doses or mRNA, BioNTech, 2 doses	30 day post-vaccination window

		BNT162b2: 39 patients. Median 55 years. (overall), 59% men (overall)			mRNA, Pfizer/Bio NTech, 2 doses or Inactivated Virus, Sinovac Biotech Ltd (CoronaVa c), 2 doses	
Slizien 2021, Poland	Longitudinal observational study	mRNA-1273 Group: 37 patients, Median 54 years, 83 men (58.45%) (overall)	Kidney, Median 8 years	Diabetes	mRNA-1273. 2 doses or BNT162b2. 2 doses	14 to 21 days after their second vaccine dose

		BNT162b2. Group (105 patients), Median 54 years, 83 men (58.45%) (overall)			BNT162b2. 2 doses or mRNA-1273. 2 doses	
Hod B 2023, Israel	Prospective cohort study.	447 patients (Humoral response subgroup: 74), Median 61.5 years, 70% Female (313/447)	Kidney, Median 4.6 years	Hypertension Diabetes Ischemic Heart Disease	BNT162b2 Vaccine. 4 doses	3 to 4 weeks (21-28 days) after the fourth booster dose .

Thomson 2022, United Kingdom	Single center cohort study	ChAdOx1(V1/2) - mRNA-1273(V3). 31 patients. Median 60 years. 65.5% male.	Kidney, duration not mentioned exactly, only association with seroconversion.	Diabetes	ChAdOx1(V1/2) - mRNA-1273(V3).<br><br>ChAdOx1(V1/2) - BNT162b2 (V3)<br><br>BNT162b2 (V1/2) - mRNA-1273(V3),<br><br>BNT162b2 (V1/2/3) (Homologous)<br><br>BNT162b2 (V1-4) (Fully Homologous),	Median 41 days after the 4th dose.

					ChAdOx1(V1/2) - BNT162b2 (V3/4)	
		ChAdOx1(V1 /2) - BNT162b2(V 3),245 patients. Median 60 years. 65.5% male.			ChAdOx1(V1/2) - BNT162b2 (V3), 3 doses	

		BNT162b2(V1/2) - mRNA-1273(V3), 25 patients. Median 60 years. 65.5% male.			BNT162b2 (V1/2) - mRNA-1273(V3), 3 doses	
		BNT162b2(V1/2/3) (Homologous), 285 patients. Median 60 years. 65.5% male.			BNT162b2 (V1/2/3) (Homologous), 3 doses	

		BNT162b2(V 1-4) (Fully Homologous), 115 patients. Median 61 years. 62.3% male.			BNT162b2 (V1-4) (Fully Homologous), 4 doses	
		ChAdOx1(V1 /2) - BNT162b2(V 3/4), 89 patients. Median 61 years. 62.3% male			BNT162b2 (V1-4) (Fully Homologous), 4 doses	

Thuluvath 2021, USA	Prospective cohort study.	BNT162b2 or mRNA-1273 or Janssen. Mean: 65.7 years, Female: 21 (34%) Male: 41 (66%)	Liver, duration not reported	Hypertension Renal impairment<br><br>Hyperlipidaemia:<br>Diabetes<br><br>Coronary artery disease Chronic obstructive pulmonary disease	BNT162b2 or mRNA-1273) or Janssen. 2 doses	4 weeks (28 days) after the final vaccine dose
Timmermann 2021, Germany	Retrospective cohort study	118 patients, Mean: 66.1 years, Male: 75 (63.6%) Female: 43 (36.4%)	Liver, Mean: 14.4 years	Alcohol-induced liver disease<br>Viral hepatitis<br>Tumour<br>Autoimmune<br>Cryptogenic<br>Other	BNT162b2. 2 doses<br><br>Or<br><br>mRNA-1273. 2 doses<br><br>Or<br><br>JNI-78436735. 1 dose	21 days after completed SARS-CoV-2 vaccination

Tsoutsoura 2023, Greece.	Prospective observational cohort study	144 patients, Mean 55.8 years, Male: 94 (65.3%) Female: 50 (34.7%)	Kidney<br><br>Heart Kidney/Heart.<br><1 year: 5 (3.5%)<br><br>1–9 years: 64 (44.4%)<br><br>More than 9 years: 75 (52.1%	Not reported	BNT162b2 or mRNA1273: Moderna. 3 doses	33 days after the administration of the third (booster)
Tylicki 2022, Poland	Longitudinal observational study	BNT162b2 Group: 60 patients, Median 54.5 years. Male: 76 (67.86%) Female: 36 (32.14%) (overall)	Kidney, Median 8 years	Not reported in detail	BNT162b2, 3 doses or mRNA-1273. 3 doses	14–21 days after the third vaccine dose

		mRNA-1273: 23 patients. Median 54.5 years, Male: 76 (67.86%) Female: 36 (32.14%) (overall)			mRNA-1273, 3 doses or BNT162b2 (Comirnaty, Pfizer/BioNTech), 3 doses	
Vaiciuniene 2021, Lithuania	Prospective observational study	136 patients, Median: 55 years, 84 Men (62.4%), 52 Women (37.6%).	Kidney,5.8 years.	Diabetes<br><br>Cardiovascular Disease<br><br>Oncological Disease	BNT162b2, 2 doses	Nine months after

Wijtvliet 2022, Belgium	Prospective, observational, multicenter study	mRNA-1273 Group: 42 patients. Sex Male: 59.5%. Median 55.5 years	Kidney, duration not reported	Diabetes Mellitus	mRNA-1273, 2 doses or BNT162b2, 2 doses	21–35 days after the second vaccine dose
		BNT162b2 Group: 91 patients<br><br>Male: 57.1%.<br>Median 57.0 years.	Kidney, duration not reported	Diabetes Mellitus	BNT162b2, 2 doses or mRNA-1273, 2 doses	

Yi 2021, USA	Prospective study - observational	145 patient, age and gender not reported	Kidney, Median 5 years.	Not reported	Pfizer-BioNTech or mRNA-1273, 1 dose for this analysis	Not reported
Zadok 2021, Israel	Prospective single-center cohort study	42 patients, Median 61 years, 83% male.	Heart, Median 110 months	Not reported	BNT162b2 . 2 doses	Day 35 to 40 after the first vaccine dose.

Rozen-Zvi 2021, Israel	Prospective cohort study	308 patients. Mean 57.51, 36%. female, 64% male.	Kidney, Mean 7.08 years.	Diabetes Mellitus.	BNT162b2. 2 doses	6 weeks after the second vaccine dose.
Midtvedt 2021, Norway	Observational study	141 patients, Median 75 years, male 56%, female 44%.	Kidney, Median 9.6 years	Not reported.	BNT162b2 . 2 doses	25–89 d after the second dose

Midtvedt 2022, Norway	Prospective observational study	188 patients, Mean 60 years, 109 (58%) Male, 79 (42%) Female.	Kidney, Mean 8.3 years.	Not reported.	Comirnaty (Pfizer-BioNTech). or Spikevax (Moderna) + Spikevax (Moderna). 4 doses	1 month after dose 4
Miele 2021, Italy.	Observational cohort study.	16 patients, Mean 57 years, 81.2% Male.	Kidney (5), Lung (5), Liver (4), Heart (2).	Not reported.	BNT162b2 mRNA. 2 doses	Median of 20 days post-vaccination

Ducloux, 2021, France	Single-center, observational study	153 patients, median 63.5 years, 60.8 male, 39.2 female.	Kidney, 158 months	Not reported.	BNT162b2. 2 doses	2.5 months after the second dose
Erol 2021, Turkey	Prospective observational study	Sinovac subgroup: 31 patients, median age 39 years, 64.5% males,	Kidney and Liver. 1-4 years: 8 patients (26.7%) 5-9 years: 10 patients (33.3%).10-14 years: 10 patients (33.3%) ≥15 years: 2 patients (6.7%)	Hypertension Diabetes	Sinovac (inactivated). 2 doses	4-6 weeks after 2nd dose

		BioNTech group: 17 patients, median age 32 years, 88.2% males	Kidney and Liver. 1-4 years: 6 patients (35.3%)<br>5-9 years: 5 patients (29.4%)<br>10-14 years: 3 patients (17.6%)<br>≥15 years: 3 patients (17.6%)	Hypertension<br>Diabetes	BNT162b2. 2 doses	4-6 weeks after 2nd dose
Haidar 2022<br>USA	Observational, prospective cohort study	450 patients, mean 61.2 years, 39.9 % female, 60.1 % male.	Kidney<br>Liver<br>Lung<br>Heart	Autoimmune/ chronic inflammatory conditions, hematological malignancies, HIV, solid tumors	mRNA-1273 or BNT162b2 or Ad26.COV2. S. 2 doses and 1 dose for Ad26.COV2. S	Not reported

Middleton 2021, United Kingdom	Retrospective cohort	698 patients. Age and sex ratio not provided.	Kidney transplant recipients only.	Not reported	COVID-19 vaccine mRNA (Pfizer-BioNTech) Or Adenovirus platform (AstraZeneca Oxford). 1 dose	Antibody testing >12 days post-vaccination

Mulder 2022. The Netherlands	Observational cohort study	476 patients, 60.1% male, median 60 years.	Liver transplant recipients	Primary sclerosing cholangitis (PSC)<br>Hepatocellular carcinoma (HCC)<br>Acute liver failure (ALF)<br>Other cholestatic diseases<br>(N)ASH<br>Cryptogenic liver disease<br>Viral hepatitis<br>Metabolic disease<br>Retransplantation (some cases)	ChAdOx1 nCoV- 19 or mRNA-1273 or BNT162b2. 2 doses	Median interval between vaccination and lab testing: 43 days for mRNA-1273 and 31 days for both BNT162b2 and ChAdOx1 nCoV-19

Nazaruk 2021, Poland	Retrospective Cohort study	61 KTRs, mean 54.4 years, 54.1 females	Kidney transplant patients. Mean 13 years.	Not reported	BNT162b2. 2 doses	4–8 weeks after the second dose
		55 LTRs, mean 58. 4 years, 32.3% females	Liver transplant patients. Mean 14.8 years	Not reported	BNT162b2. 2 doses	

Correia 2022, Portugal	Single-center, prospective, observational study	mRNA group: 70 patients, mean age 58.5 years, 60% males	Kidney transplant recipients, >6 months post-transplant.	Diabetes Mellitus High Blood Pressure Obesity	BNT162b2 or mRNA-1273. 2 doses	Anti-spike IgG antibodies were evaluated at a mean time of 20 days post-vaccination conclusion
	\	Adenovirus group: 61 patients, mean age 60.2 years, 70. 5% males			ChAdOx1-S, AstraZeneca (2 doses) or Janssen, Ad26.CoV2. S, Johnson & Johansons (1 dose)	

Prendecki 2021, United Kingdom	Prospective observational cohort study.	Previous infection: 152 patients. Age and sex not specified	Kidney transplant recipients, duration not specified	Diabetes	BNT162b2 or ChAdOx1. 2 doses	median of 31 days after vaccination
		Infection naïve patients, BNT162b2 group: 410 patients. Age and sex not specified			BNT162b2. 2 doses	

		Infection naïve patients, ChAdOx1 group: 358 patients. Age and sex not specified			ChAdOx1. 2 doses	
Saharia, 2022, United States	Prospective cohort study	53 patients, Median age: 64 years, 71.7% male.	Kidney, Liver, Lung, Heart, Kidney/Pancreas, Heart/Lung, Liver/Kidney, Pancreas.	Diabetes, Obesity, Chronic Kidney Disease, Pulmonary Disease, Heart Failure Myocardial Infarction	Pfizer or Moderna. 3 doses	For the post-booster analysis, samples were taken ≥2 weeks after the third dose.

Sakai 2022, Japan	Observational study	56 patients, Median age 65.0 years, 76.8% male	Liver transplant recipients only. Median duration 15.5	Not reported	BNT162b2. 2 doses	Blood was collected at least 14 days after the second vaccination .
Spinner 2022, United States	Retrospective, single-institutional cohort study	40 patients, median age 17.1, 27 males, 13 females.	Pediatric Heart Transplant (HTx) recipients. Duration not reported	Chronic Kidney Disease, Diabetes Mellitus, and BMI categories (overweight, obese).	Pfizer–BioNTech BNT162b2 (2-dose series) or Moderna mRNA-1273 (2-dose series) or Johnson & Johnson/Janssen Ad26.COV2. S (single dose)	Median 229 days.

Strauss 2021, USA	Prospective observational cohort study	161 patients; median age 64 years, 57% female, 43% male	Liver transplant recipients only; median time since transplant 6.9 years	Not reported	BNT162b2 or mRNA-1273., 2 dose series	Median of 30 days after D2
Toniutto, 2022, Italy	Prospective cohort study	Covid 19 naïve patients: 131 patients, median age 57.9 years, 70.2% male	Liver transplant, months between LT and vaccination: median 94 months	Diabetes Mellitus Dyslipidemia Arterial Hypertension Hepatocellular Carcinoma Recurrent cirrhosis with esophageal	BNT162b2 mRNA vaccine, 2 doses.	Mean 165 days (approx. 6 months)

		Covid 19 recovered patients: 12 patients, median age: 57.5 years, 83.3% male	Liver transplant, months between LT and vaccination: median 157 months.	varices Ascites	BNT162b2 mRNA vaccine, 2 doses.	
Yanis 2021, USA	Prospective cohort study	56 SOT recipients, mean age 72.1. 61.1% male	Kidney, Liver, Heart, Lung, Kidney Liver. Median time post-transplant: 7.2 years.	Hypertension Dyslipidemia chronic kidney disease cardiovascular disease diabetes mellitus obesity chronic lung disease autoimmune disease malignancy chronic liver disease	BNT162b2. 2 doses	21–42 days after second dose

SOT: Solid Organ Transplant; KTR: Kidney Transplant Recipient; LTR: Liver Transplant Recipient; HTR: Heart Transplant Recipient; MMF: Mycophenolate Mofetil; MPA: Mycophenolic Acid; BMI: Body Mass Index; eGFR: Estimated Glomerular Filtration Rate;

COPD: Chronic Obstructive Pulmonary Disease; ILD: Interstitial Lung Disease; CF: Cystic Fibrosis; RCT: Randomized Controlled Trial; HTN: Hypertension; CVD: Cardiovascular Disease; CKD: Chronic Kidney Disease; DM: Diabetes Mellitus.

Electronic Supplementary Table 3. Efficacy and immunogenicity of COVID-19 vaccines amongst the included studies.

Study (Author, Year)	Population Analyzed (N)	Assay type	Baseline COVID-19 status	Seroconversion definition	Seroconversion Rate n/N (%) *	Geometric Mean/Median Titer [95% CI/IQR]	Vaccine Effectiveness	Key Findings / Notes
Azzi 2021, USA	No prior Covid group: 76 patients	SARS-CoV-2 spike IgG (Anti-spike IgG) - Specific assay not named.	All patients were seronegative	Presence of anti-spike IgG after vaccination.	24 / 76 (32%)	Not reported	Not reported	
	Prior Covid group: 21 patients		Patients had prior infection		20 / 21 (95%)			
Cholankeril, 2022, USA	69 patients	Laboratory-based immunoassays (Quest Diagnostics) for antibodies to the S1 domain SARS-CoV-2 spike protein and nucleocapsid protein.	All patients were seronegative	Spike immunoglobulin G (IgG) titre ≥1.00.	33 / 69 (48%)	Not reported	Not reported	

Westhoff 2021, Germany	10 patients	ELISA (for S-protein-binding antibodies), Pseudovirus system (for neutralizing antibodies).	All patients were seronegative	Development of measurable SARS-CoV-2 spike antibodies	6/10 (60%)	Not reported	Not reported	
D'Offizi, 2021, Italy	61 patients	ARCHITECT® chemiluminescence microparticle antibody assay (Abbott) for anti-spike IgG.	All patients were seronegative	Anti-spike IgG ≥7.2 Binding Arbitrary Units (BAU)/ml.	47 / 61 (77%)	Not reported	Not reported	
Sadioglu, 2021, Turkey	85 patients	COVID-19 IgG – ELISA (DIA.PRO)	All patients were seronegative	Anti-SARS-CoV-2 IgG level >10 IU/mL.	16 / 85 (18.8%)	Not reported	Not reported	
Sattler, 2021, Germany	39 patients	ELISA for spike S1 domain-specific IgG and IgA (EUROIMMUN Surrogate virus neutralization test (sVNT, GenScript).	All patients were seronegative	OD ratio >1.1 per manufacturer's guidelines (EUROIMMUN ELISA).	1/39 (2.6%)	Not reported	Not reported	

Davidov, 2021, Israel	76 patients	Enzyme-linked immunosorbent assay (ELISA) for IgG antibodies against the RBD.	All patients were seronegative	IgG antibody titres ≥1.1 sample-to-cutoff ratio.	55 / 76 (72%)	2.1 (95% CI, 1.6–2.6)	Not reported	
Rashidi-Alavijeh 2021, Germany	43 patients	LIAISON® SARS-CoV-2 TrimericS IgG CLIA (Diasorin).	All patients were seronegative	Values ≥13.0 AU/mL were considered positive.	34/43 (79%)	Not reported	N/A	LT recipients showed weaker responses than controls but stronger than other transplants; median titers 216 in patients vs >2080 BAU/mL in controls.

Rincon-Arevalo 2021, Germany	40 patients	Euroimmun ELISA (anti-S1 IgG/IgA, anti-NCP IgG)<br><br>GenScript surrogate virus neutralization test (sVNT)<br><br>Flow cytometry Single-cell RNA-seq: CITE-seq	1 patient identified via anti-NCP antibodies.	A positive anti-S1 IgG result according to the Euroimmun ELISA after their second vaccine dose.	1/40 (2.5%)	Not reported	N/A	.
Balsby 2022, Denmark	358 patients	Abbott SARS-CoV-2 IgG II Quant (cutoff: 7.1 BAU/mL)<br><br>Meso Scale Diagnostics assay (cutoff: 27.2 BAU/mL)	All patients were seronegative	Defined as having an antibody concentration above the assay cut-off.	275/358 (77%)	Not reported	Breakthrough infection SOT: 40% (150/377)	Third mRNA dose raised seroconversion in SOT recipients from 49% to 77%, still below controls (99%).

Bergman 2021, Sweden	83 SOT	Roche Elecsys Anti-SARS-CoV-2 S (anti-RBD IgG).	1/89 also RT-PCR positive.	Anti-SARS-CoV-2 spike RBD ≥0.8 U/mL, 14 days post-second dose.	36/83 (43.4)	Not reported	1 in SOT patients	Among immunocompromised patients. Worst responders: SOT recipients (43.4%)
Benotmane 2021 A, France	204 patients	ARCHITECT IgG II Quant test (Abbott) for anti-spike IgG.	All patients were seronegative	Antibody titer >50 AU/mL.	98/204 (48%)	Not reported	1 patient developed a severe form of COVID-19 five days after the second injection.	Two doses of an mRNA vaccine provide insufficient protection for a large proportion of KTRs
Marion, 2021, France	Kidney: 271patients	SARS-CoV-2 total antibodies enzyme-linked immunosorbent assay test (Beijing Wantai Biological Pharmacy Enterprise)	5 patients	Positive antibody test result post-vaccination	89 / 271 (33%)	Not reported	No breakthrough infections	
	Liver: 58 patients				29 / 58 (50%)			
	Thoracic organs: 12 patients				4 / 33 (12%)			

	Pancreas: 5 patients				1/5 (20%)			
Kamar 2021, France	101 patients	Wantai enzyme-linked immunosorbent assay (ELISA) for antibodies to SARS-CoV-2 spike protein.	All patients were seronegative	Presence of anti-SARS-CoV-2 antibodies (positive signal-to-cutoff ratio).	67 / 99 (68%)	Not reported	No breakthrough infections	
Del Bello 2022, France	396 patients	Wantai enzyme-linked immunosorbent assay (ELISA) test<br><br>Anti- SARS-CoV- 2 spike protein assay.	1.3% (n=5) had anti-SARS-CoV-2 antibodies before the first vaccine dose.	A positive test using the Wantai assay was defined by a signal- to- cut- off ratio (S/CO) >1.1.	269/396 (67.9%)	Not reported	Not reported	Antibody levels rose sharply after the third dose, especially in previously seropositive patients.<br><br>269 patients were analyzed for the third dose

Benotmane 2021 B, France	242 patients	ARCHITECT IgG II Quant test (Abbott) for anti-spike IgG.	All patients were seronegative	Positive anti-SARS-CoV-2 spike antibody response, defined as >50 AU/mL at 28 days post-first vaccine dose.	26/241 (10.8%)	Not reported	1 KTR developed mild symptomatic COVID-19 7 days post-injection.	The study advises against delaying the second dose and supports additional boosters for protection.
Benotmane 2021 C. France	159 patients	ARCHITECT IgG II Quant test (Abbott) for Anti-receptor-binding domain (RBD) IgG.	All patients were seronegative	Anti-RBD IgG titer >50 AU/mL.	78 / 159 (49%)	Not reported	Not reported	
Cao 2022, USA	37 patients	Abbott Alinity for anti-S IgG; Enzyme-linked immunosorbent assay (ELISA) for neutralization.	All patients were seronegative	Defined as anti-S IgG ≥50 AU/mL.	15/37 (40.5%)	Not reported	1 SOT recipient identified via PCR screening 13 days after complete vaccination.	Median antibody levels in SOT recipients were ~10,000x lower than in healthy vaccinated people.

Cotugno 2022, Italy	34 patients.	Anti-SARS-CoV-2 S1 RBD antibody (Roche); Anti-trimeric SARS-CoV-2 antibody (LIAISON SARS-CoV-2, DiaSorin); Flow cytometry for T-cell and B-cell phenotyping.	No history of COVID-19.	Defined as the development of detectable SARS-CoV-2 immunoglobulin G (IgG) antibodies following vaccination.	24/ 34 (71%)	Not reported	Not reported	Anti-SARS-CoV-2 S1 RBD Antibody: Significantly lower in SOTs vs. Healthy Controls (HCs) at T21 and T28 ($P < 0.0001$).
Devresse 2021, Belgium	90 patients	Elecsys anti-SARS-CoV-2 immunoassay (Roche) against spike RBD.<br><br>Whole blood interferon-gamma release assay (IGRA, Euroimmun) using SARS-CoV-2 spike protein antigens.	7 patients had a previous documented SARS-CoV-2 infection before vaccination.	Anti-RBD antibody titer >0.8 U/mL by Elecsys assay (Roche).	58/90 (64.4%)	Not reported	Not reported	Humoral response: 64%; cellular response: 32%.<br><br>Strong antibody response (>150 U/mL) correlated with strong T-cell response.

Firket 2021, Belgium	10 patients with prior Covid 19	DiaSorin LIAISON® chemiluminescence immunoassay (quantifying anti-S1/S2 IgG)	20 with prior COVID-19 (10 KTRs, 10 controls) and 20 without (10 KTRs, 10 controls).	Defined as detectable anti-S1/S2 IgG by DiaSorin LIAISON® assay	10/10 (100%) .	Not reported	N.A	Prior COVID-19: 100% seroconverted after first dose.
	10 patients without prior Covid 19	DiaSorin LIAISON® chemiluminescence immunoassay (quantifying anti-S1/S2 IgG)	20 with prior COVID-19 (10 KTRs, 10 controls) and 20 without (10 KTRs, 10 controls).	Defined as detectable anti-S1/S2 IgG by DiaSorin LIAISON® assay.	3/10 (30%)	Not reported	N/A	No prior COVID-19: 10% after second dose, rising to 30% weeks later
Georgery 2021, Belgium.	79 patients	Elecsys anti-SARS-CoV-2 immunoassay (Roche Diagnostics GmbH).	All patients were seronegative	Positive anti-SARS-CoV-2 antibody: >0.8 U/mL at 28 days post-second dose.	39/79 (49%)	Not reported	Not reported	Low response: 49% of KTRs seroconverted after two Pfizer doses; 90% had intermediate titers, and only 10% achieved high titers (>250 U/mL).

Masset 2021, France	456 patients	Chemiluminescent microparticle immunoassay (Abbott Architect), chemiluminescence immunoassay (Siemens Atellica), electrochemiluminescence immunoassay (Roche Elecsys)	All patients were seronegative	Anti-spike IgG level above laboratory threshold	94/136 (69.2%)	Not reported	Not reported	
Griessbach 2022, Switzerland	26 patients	Roche Elecsys Anti-SARS-CoV-2 S; ABCORA 2	All patients were seronegative	Primary outcome: proportion of patients with anti–SARS-CoV-2 S1-RBD antibodies ≥100 U/mL (Elecsys, Roche)	17/26 (65.4%)	Not reported	Not reported for SOT patient	Third-dose mRNA vaccination improved seroconversion in SOT recipients from 23.9% (after 2 doses) to 65.4%.

Havlin 2022, Czech Republic	15 patients	Microblot-Array COVID-19 IgG (TestLine);<br><br>Intracellular cytokine staining by flow cytometry.	All patients were seronegative	Detection of anti-SARS-CoV-2 IgG against recombinant antigens via Microblot-Array COVID-19 IgG test.	2/15 (13%)	Not reported	N/A	
Hoffman 2021, The Netherlands	No COVID-19 before vaccination: n=79	Liaison platform (DiaSorin) for SARS-CoV-2 spike S1/S2 protein-specific IgG antibodies	12 patients had COVID-19 prior to vaccination; 80 patients did not.	Defined as IgG > assay's 2.5th percentile of healthy controls post-vaccination. .	21/79 (27%)	Not reported	N/A	mRNA-1273 (Moderna) vaccine (n = 89). BNT162b2 (Pfizer–BioNTech) vaccine (n = 2)
	Had COVID-19 before vaccination: n=12				9/12 (75%)	Not reported	N/A	

Holden 2021, Denmark	80 patients	EUROIMMUN SARS-CoV-2 ELISA (IgG, S1, FDA-approved);<br><br>IFN-γ ELISpot (Qiagen QuantiFERON).	All patients were seronegative	Positive S1 IgG response: ratio >0.8 at 6 weeks post–second dose.	28/79 (35%)	Not reported	N/A	
Husain 2021. USA	Moderna group: 12 patients	Clinical anti-spike IgG immunoassays (Liaison [DiaSorin] or Elecsys [Roche])	3 patients (11%) had prior PCR-confirmed SARS-CoV-2 infection. (vaccine group not specified)	Anti-spike IgG measured 2–6 weeks post–second dose; positive if above assay threshold.	3/12 (25%)	Not reported	N/A	Finding highlights that transplant recipients, who are at high risk for severe COVID-19, may remain vulnerable even after full vaccination.<br>.
	Pfizer group: 16 patients.				4/16 (25%)			

Marinaki 2021, Greece	34 patients	Chemiluminescent Microparticle Immunoassay (CMIA), Abbott SARS-CoV-2 IgG II Quant (against RBD)	Not stated	An anti-SARS-CoV-2 RBD IgG concentration ≥50 AU/ml measured after the second vaccine dose.	20/34 (58.8%)	Not reported	N/A	The use of antimetabolite immunosuppressants (like mycophenolate) was the strongest predictor of a poor vaccine response.
Chavarot 2021, France A	101 patients	SARS-CoV-2 IgG II Quant (Abbott) or Wantai ELISA. T-cell: IFN-γ ELISpot assay.	Patients were seronegative	Defined per manufacturer criteria as a positive anti–SARS-CoV-2 spike antibody test.	2/35 (5.7%)	Not reported	N/A	
Chavarot 2021, France B	COVID-19 naïve: 97 patients	SARS-CoV-2 IgG II Quant (Abbott) anti-spike protein	No prior infection in main cohort (n=97)	A positive response was defined as anti–SARS-CoV-2 spike IgG >50 AU/mL (Abbott IgG II Quant, USA).	17/97 (17.5%)	Not reported	Breakthrough infections Not reported	
	Prior COVID-19: 5 patients.		Prior infection in subgroup (n=5)		5/5 (100%)	Not reported		

Noble 2021, France	57 patients	Enzyme immunoassay against the S1 domain of the SARS-CoV-2 spike protein (Wantai Biological Pharmacy Enterprise).	All patients were seronegative	A positive immune response was defined as anti-SARS-CoV-2 S1 antibodies detected by the Wantai ELISA.	21/57 (36%)	Exact number not reported	Not reported	Only 20 patients were assessed at the 3rd dose.
Pedersen 2021, Denmark	58 patients	Plaque Reduction Neutralization Test (PRNT) with live SARS-CoV-2.<br><br>Ortho CD VITROS Anti–SARS-CoV-2 IgG & Diasorin Liaison SARS-CoV-2 TrimericS IgG.	All patients were seronegative	Defined as an antibody rise from negative to >17.8 BAU/mL on the Vitros assay.	18/58 (31%)	Not reported	Not reported	.

Medina-Pestana 2022, Brazil.	3354 patients	AdviseDx SARS-CoV-2 IgG II assay (Abbott Laboratories).	All patients were seronegative	Defined as an antibody level >50 arbitrary units per milliliter (AU/mL)	143/942 (15%)	Not reported	61 (1.8%) patients had confirmed COVID-19 after the first dose.	Poor protection: 1.8% of vaccinated patients got COVID-19; of these, 72% were hospitalized and 26% died, similar to unvaccinated rates.
Shostak 2021, Israel	168 patients	SARS-CoV-2 IgG II Quant assay (Abbott Ireland Diagnostic Division) for quantitative measurement of anti-spike IgG (S-IgG).	All patients were seronegative	Seroconversion is defined as anti-spike IgG ≥50 AU/mL.	31/168 (18%)	Not reported	No breakthrough infections	168 patients were included in the final analysis

Stumpf 2021 A, Germany	71 patients	ELISA for IgA/IgG against spike S1 protein, IgG against RBD.<br><br>Interferon-γ release assay (IGRA).	Patients were SARS-CoV-2 naïve	Defined as de novo antibody development post-vaccination exceeding predefined thresholds for anti-spike S1 or anti-RBD IgG/IgA.	39/71 (55%)	Not reported	N/A	Humoral response rose from 32% (two doses) to 55% (three doses). Neutralizing RBD antibodies reached 94% after the third dose.

Stumpf 2021 B, Germany	368 patients (99 patients for BNT162b2)	Euroimmun ELISA (Anti-Spike S1 IgG/IgA, Anti-NCP IgG, Anti-RBD IgG). Interferon-γ Release Assay (IGRA) and Flow Cytometry (FACS).	Patients were SARS-CoV-2 naïve	Defined as de novo antibody development (seroconversion) at T1 or T2	26/99 (26%)	Not reported	Symptomatic: 4/376 (1.1%) Asymptomatic: 4/376 (1.1%)	Vaccine type was an independent risk factor for seroconversion failure, with BNT162b2 linked to higher odds of non-response despite adjustment for age, sex, and immunosuppression
	368 patients (234 for mRNA-1273)				115/ 234 (49%)			In KTRs, mRNA-1273 (Moderna) induced stronger humoral and cellular responses than BNT162b2 (Pfizer/BioNTech).

Villanego 2021, Spain	91 patients	Abbott SARS-CoV-2 IgG chemiluminescent microparticle immunoassay	6 patients had prior COVID-19.	A positive result on the Abbott SARS-CoV-2 IgG assay in a patient who was seronegative before vaccination.	57/91 (62.6%)	Not reported	Breakthrough infections occurred in 15/ 843 vaccinated patients	Patients with prior COVID-19 had a 20-fold increase (201.8 vs. 3601.2 U/ml).
Benning 2022, Germany.	40 patients	Siemens SARS-CoV-2 Total Assay<br>One Lambda LabScreen Covid Plus<br>Medac surrogate neutralizing antibody (snAB) test<br>Roche Elecsys assay<br>Live-Virus Neutralization Assay	Patients had no prior Covid 19	Positive antibody assays.<br>Siemens, index ≥1<br>Medac, ≥30% RBD: ACE-2 inhibition	28/40 (70%)	Not reported	12/49 (25%) occurred a median of 5.2 months after the third dose. 11/12 occurred during the Omicron surge.	
	7 patients				6/7 (86%)			
	2 patients				1/2 (50%)			

Bertrand 2021 A, France	45 patients	Humoral: ARCHITECT IgG II Quant test (Abbott) for Anti-spike IgG. Cellular: IFNγ ELISPOT assay for T-cell response	All patients were seronegative at baseline	Anti-spike IgG titre >50 AU/mL.	8 / 45 (17.8%)	Not reported	Not reported	
Bertrand 2021 B, France	80 patients	Humoral: Anti-spike IgG assay (specific test not named, threshold: 50 AU/ml). Cellular: IFNγ ELISPOT assay for T-cell response.	All patients were seronegative at baseline	Anti-spike IgG titre >50 AU/mL.	49 / 80 (61.2%)	Not reported	2 breakthrough infections	

Bertrand 2021 C, France	235 patients.	ARCHITECT IgG II Quant (Abbott Laboratories)	All patients were seronegative	Anti-spike IgG >50 AU/mL (Abbott ARCHITECT IgG II Quant).	65/235 (27.7%)	Not reported	Breakthrough gh infection 3.4% (8/ 235). Group not specified	
Boyarsky 2021 A, United States	Janssen vaccine group 12 participants.	Roche Elecsys anti-SARS-CoV-2 S enzyme immunoassay (Target: anti-RBD antibodies).	All patients were seronegative .	Detectable anti-RBD antibodies against SARS-CoV-2 spike protein by Roche Elecsys assay.	2/12 (17%)	Not reported	N/A	Analysis excluded mRNA recipients >70 years old to match the Janssen group's baseline.
	mRNA vaccine group: 725 participants				30/725 (59%)	Not reported	N/A	
Boyarsky, 2021 B, USA	436 patients	EUROIMMUN ELISA (anti-S1) or Roche Elecsys (anti-RBD)	All patients were seronegative	Detection of antibody (anti-S1 or anti-receptor-binding domain) post-vaccination.	76/436 (17.4%)	Not reported	N/A	

Boyarsky, 2021 C USA	658 patients	Roche Elecsys (anti-RBD) or EUROIMMU N ELISA (anti-S1)	All patients were seronegative	Detection of antibody (anti-RBD or anti-S1) post-vaccination using manufacturer cutoffs (Roche: ≥0.8 U/mL; EUROIMMUN: ≥1.1 arbitrary units).	357/658 (54.3%)	Not reported	N/A	
Brandstetter 2022, Austria	324 patients	SARS-CoV-2 IgG II Quant assay (Abbott); results in BAU/ml.	Patients were seronegative	Defined as having an anti-spike-RBD-IgG antibody titer of <7.1 Binding Antibody Units per milliliter (BAU/ml).	12/41 (29.3%)	Not reported	Breakthrou gh infections 4 cases: 1 after 2 doses (asymptom atic), 3 after 3 doses (2 hospitalize d).	After dose 3: 147 patients completed follow-up (157 seronegative after dose 2, with 10 dropouts).<br><br>After dose 4: 41 patients completed follow-up (53 seronegative after dose 3, with 12 dropouts).

Bruminhent 2022, Thailand	mRNA vaccine: 40 patients	SARS- CoV- 2 NeutraLISA surrogate neutralization test assay (Euroimmun)	All patients were seronegative	Defined as anti-RBD IgG ≥7.1 BAU/mL.	mRNA Group: 28/40 (70%)	Not reported	N/A	In kidney transplant recipients, mRNA and viral vector boosters elicited similar immunogenicity.
	Viral vector vaccine group (V group): 37 patients		All patients were seronegative	Defined as anti-RBD IgG ≥7.1 BAU/mL.	Viral Vector Group: 24/37 (65%)		N/A	No significant differences were seen between vaccine platforms in seroconversion, antibody levels, neutralization, or T/B-cell responses.

Buchwinkler 2021, Austria	216 patients	Abbott SARS-CoV-2 IgG II Quant (40.7%) Liaison® SARS-CoV-2 S1/S2 IgG (59.3%)	. All patients were seronegative	Defined as a positive anti-spike antibody titer above the manufacturer's cutoff.	108/216 (50%)	Not reported	Breakthrough infection not analyzed	Half of KTRs lacked response after two mRNA doses, with lower titers than CKD or HD patients.
Chukwu 2022, United Kingdom	373 patients, BNT162b2: 216 patients	Siemens Atellica-IM SARS-CoV-2 immunoassay (anti-S1 RBD antibodies)	All patients were seronegative	A positive anti-SARS-CoV-2 antibody test result (index value >1.0).	114/216 (52.8%)		Total post-vaccination infections: 22/373 (5.9%) Non-responders: 17 infections (77%) Responders: 5 infections (23%)	
	373 patients, AZD1222: 84 patients				50/84 (59.5%)			

Crane 2021, USA	25 patients	Abbott chemiluminescent microparticle immunoassay (positive >50 AU/mL) Siemens Atellica IM SARS-CoV-2 IgG (positive >1.0 index)	3 patients (12%) had a prior symptomatic COVID-19 infection.	Antibody titer greater than 50 AU/mL for the Abbott assay or greater than 1.0 index for the Siemens assay.	13/25 (52%)	Not reported	No breakthrough infections	25 (30 received two doses, but only 25 had antibody titers drawn and were analyzed).
Crespo 2021, Spain	90 patients	LIAISON® SARS-CoV-2 TrimericS IgG kit (Diasorin Inc.) QuantiFERON® SARS-CoV-2 IFNγ release assay (IGRA) (QIAGEN)	No prior Covid 19 infection	When antibodies first appear in the blood, showing an immune response.	57/90 (63.3%)		N/A	

Cucchiari 2021, Spain	148 patients	Luminex (IgG/IgM against RBD) IFN-γ ELISpot (S and N proteins)	SARS-CoV-2-naïve: 117 (79.1%) SARS-CoV-2-pre-immunized: 31 (20.9%)	Defined as developing detectable IgM or IgG antibodies against the spike protein 2 weeks after the second vaccine dose.	35/ 117 (29.9%)	Not reported	No breakthrough infections	Only 29.9% of naïve patients developed humoral immunity. Cellular response (54.7%) increased overall immunogenicity to 65%.
Danthu 2021, France	74 patients	Antibodies: LIAISON SARS-CoV-2 TrimericS IgG (DiaSorin) Anti-N antibodies: Abbott Alinity (to exclude prior infection)	All patients were seronegative	Defined as an anti-spike SARS-CoV-2 antibody titer >13 AU/mL	3/74 (4.1%)	Not reported	No breakthrough infections	Median titer not calculable (only 3 responders) for KTR patients

Dov 2022, Israel	252.patients	LIAISON SARS-CoV-2 S1/S2 IgG (DiaSorin). And ARCHITECT SARS-CoV-2 IgG II (Abbott).	Prior infection rates: KTR: 9.1%.	Antibody titers ≥19 AU/ml.	82/195 (42%)	Not reported	Not reported	KTRs had poor seroconversion (42%) compared to dialysis (79%) and controls (100%).<br><br>Immunosuppression (tacrolimus/mTOR inhibitors) strongly predicted non-response.

Fernández-Ruiz,<br>2021, Spain	44 patients	ELISA (EUROIMMUN Anti-SARS-CoV-2 IgG). In-house hACE-2/spike antibody inhibition ELISA.	1 patient but 3 patients had unrecognized natural infection	Detected using the EUROIMMUN Anti-SARS-CoV-2 ELISA. Positive (Seroconverted): Ratio ≥1.1	24/42 (57.1%)	Not reported	1 patient had a breakthrough infection	SARS-CoV-2-specific Cell-mediated Immunity (CMI): 59.5% (25/42 patients)<br><br>Serum Neutralizing Activity: 31.0% (13/42 patients) 42 patients completed the analysis
Frolke 2022, The Netherlands<br>.	2,092 patients<br>1401 patients	Sanquin anti-SARS-CoV-2 RBD IgG ELISA. Fluorescent bead-based multiplex immunoassay (RIVM).	No history of SARS-CoV-2 infection	Defined as having anti-spike IgG antibody levels ≥50 BAU/mL (in most cohorts)	1132/2092 (54.1%)<br>1043 / 1401 (74.4%)	Exact number not given	N/A	

Grupper 2021, Israel	136 patients	LIAISON SARS-CoV-2 S1/S2 IgG chemiluminescent assay (DiaSorin).	All patients were seronegative	IgG anti-spike antibody level >15.0 AU/mL.	51/ 136 (37.5%)	Not reported	2 cases in the seronegative KTR group during the 3-month follow-up.	
Guarino 2022, Italy	No Prior COVID): 444 patients	LIAISON® SARS-CoV-2 S1/S2 IgG (DiaSorin).	Previous Covid 19 infection: 48 patients	Anti-Spike IgG >25 AU/mL by the LIAISON S1/S2 IgG assay.	336/444 (75.67%)		3 out of 492 patients (0.6%) breakthrough infections	
	Previous COVID-19: 48 patients				42/48 (87.5%)			
Hall 2021 A, Canada	127 patients	Anti-RBD: Roche Elecsys Neutralization: GenScript SVNT T cells: Flow cytometry	All the main cohort was seronegative, natural infection cohort was used as a comparator	A positive anti-RBD antibody titer of ≥0.8 U/mL.	38/110 (34.5%)	Not reported	2/127 (1.57%; 1 fatal).	Patients who Completed: 110 (provided serum at all time points).

Hall 2021 B, Canada	60 patients	Elecsys Anti-SARS-CoV-2 immunoassay (Roche) for anti-RBD antibodies.	All patients were seronegative	Anti-receptor-binding domain (RBD) antibody level of at least 100 U per millilitre.	33 / 60 (55%)	Not reported	Not reported	
Hallet 2021, USA	Heart transplant recipient: 134 patients	Roche Elecsys Anti-SARS-CoV-2 (Anti-RBD) and EUROIMMUN Anti-SARS-CoV-2 ELISA (Anti-S1).	All patients were seronegative	Defined as a positive anti-spike antibody result: Roche Elecsys ≥0.80 U/mL or EUROIMMUN ≥1.1 AU	64/134 (48%)	Not reported	N/A	
	Lung Transplant (LT) Recipients: 103 patients				28/103 (27%)			
Hamm 2022, Denmark	200 patients	Anti-RBD IgG: In-house ELISA. In-house ELISA (ACE-II/RBD inhibition)	All patients were seronegative	Defined as having both anti-RBD IgG >225 AU/ml AND neutralizing capacity >25%.	72 / 200 (36%).	Not reported	5 /156 SOT recipients had PCR-confirmed SARS-CoV-2 infection.	

Harberts 2022, Germany	106 patients	Roche Elecsys anti-SARS-CoV-2 S (RBD) assay. Interferon-gamma release assay (IGRA, EURO-IMMUN) and a sensitive in vitro T-cell expansion assay	No prior infection	Defined as a detectable response (≥ 0.8 AU/mL) after any vaccination in LTRs or HCs who previously showed no response (<0.8 AU/mL).	97/106 (92%)	Not reported	Not reported	
Haskin 2021, Israel	38 patients	SARS-CoV-2 IgG II Quant assay (Abbott) against the spike protein.	No prior Covid 19 infection	A positive serologic response was defined as SARS-CoV-2 spike IgG >50 AU/mL (Abbott IgG II Quant).	24/38 (63%)	Not reported	N/A	

Havlin 2021, Czech Republic	LTRs patients post-COVID n=33.	ELISA: Anti-SARS-CoV-2 Spike S1 IgG (Euroimmun) Microblot-Array: COVID-19 IgG (TestLine) CLIA: Liaison SARS-CoV-2 Trimeric S IgG (Diasorin)	Post-COVID Group: All had prior PCR-confirmed infection.	A detectable anti-SARS-CoV-2 Spike IgG level above the positivity threshold, measured by ELISA, Microblot-Array, or CLIA.	17/21 (81%)	Not reported	3 patients diagnosed with COVID-19 post-vaccination	Infection > Vaccination: 85% of LTRs developed antibodies after natural COVID-19 infection, highlighting a stark contrast.
	LTRs Vaccinated patient: 48 patients		No history of infection (seronegative at baseline).		0/21 (0%)	Not reported	N/A	
Heinzel 2022, Austria	Homologous (mRNA) Group: 85 patients	Roche Elecsys anti-SARS-CoV-2 S electrochemiluminescence immunoassay (ECLIA).	No history of infection (seronegative at baseline).	An antibody level > 0.8 U/mL as measured by the Roche Elecsys anti-SARS-CoV-2 S enzyme immunoassay	38/85 (45%)	Not reported	N/A	
	Heterologous (Vector) Group: 84 patients				42/84 (50%)			

Herrera 2021, Spain	Liver Transplant Recipients: 58 patients	Siemens Atellica IM SARS-CoV-2 Total (COV2T) and IgG (COV2G) chemiluminescent immunoassays	All patients were seronegative	Positive test for SARS-CoV-2 S1-RBD IgM/IgG antibodies 4 weeks after receiving the second dose of the vaccine.	41/58 (71%)	Not reported	Not reported	
	Heart Transplant Recipients: 46 patients				26/46 (57%)			
Hod A 2021, Israel	120 patients	In-house ELISA. SARS-CoV-2 pseudo-virus neutralization assay.	All patients were seronegative	Defined as RBD IgG ≥1.1 and the presence of NA capable of reducing viral replication by 50% at a 16-fold dilution or above.	42/120 (35%)	Not reported	Not reported	

Kantauskaite 2021, Germany	225 patients	Anti-SARS-CoV-2-QuantiVac-ELISA (IgG against spike S1 subunit) Live virus neutralization test using infectious SARS-CoV-2 B.1 isolate	All patients were seronegative	Measured by the presence of IgG antibodies against the SARS-CoV-2 spike S1 subunit at a level >35.2 BAU/mL.	56/225 (24.9%)	Not reported	N/A	
Karaba 2022, United States	25 patients.	Meso Scale Diagnostics: Anti-RBD seropositivity	1 patient	Anti-RBD and/or anti-spike IgG positive based on the manufacturer's cutoff.	21/25 (84%)	Not reported	N/A	

Kho 2022, The Netherlands.	Group 1A: Single Dose mRNA-1273 (Control Group), 73 patients randomized	Plaque reduction neutralization test (PRNT). Validated fluorescent bead-based multiplex.	No prior or current diagnosis of COVID-19	Achieved S1-specific IgG ≥10 BAU/mL at 28 days post-vaccination.	50/73 (68.5%)	Not reported	1 COVID-19 infection reported during the study.	74 completed day 28
	Group 1B: Double Dose mRNA-1273, 72 patients randomized				49/72 (68.1%)		2 COVID-19 infections reported during the study.	75 completed day 28
	Group 1C: Heterologous Ad26.COV2-S, 73 randomized				46/73 (63%)		3 COVID-19 infections reported during the study.	75 completed day 28

Korth 2021, Germany	23 patients	Anti-SARS-CoV-2 IgG CLIA (LIAISON® SARS-CoV-2 TrimericS IgG assay)	No prior or current diagnosis of COVID-19	A positive anti-SARS-CoV-2 IgG result (≥13.0 AU/mL) against the spike protein using the LIAISON® TrimericS IgG CLIA assay.	5/23 (22%)	Not reported	N/A	
Kumar 2022, Canada	60 patients	SARS-CoV-2 spike pseudo typed lentivirus neutralization assay.	No prior or current diagnosis of COVID-19	Defined as having a detectable anti-receptor binding domain (RBD) antibody titer ≥0.8 U/ml.	39/51 (76.5%)	Not reported	N/A	
Marlet 2021, France.	160 patients	SARS-CoV-2 IgG II Quant assay (Abbott Alinity i) Commercial.	7 patients	Anti-spike IgG ≥7.1 BAU/mL (manufacturer's cut-off), with ≥30 BAU/mL used as a stricter threshold linked to 50% vaccine effectiveness.	≥7.1 BAU/mL: 47% (75/160)	Not reported	N/A	

Massa 2021, France.	61 patients	ELISA (ABBOTT) for anti-RBD IgG. Pseudo-neutralization assay (MSD) inhibiting Spike-ACE2 binding	All patients were seronegative	Conversion from negativity (i.e., ≤ 50 AU/mL) to positivity (i.e., > 50 AU/mL).	38/61 (62.3%)	Not reported	N/A	
Masset 2022, Germany	Heterologous Vaccination Group: 28 patients	Anti-spike IgG quantified by ECLIA (Roche), Architect (Abbott), or Diasorin. Expressed in Binding Antibody Units (BAU)/ml.	All patients were seronegative	Detection of anti-spike IgG above the lab's positivity threshold after vaccination.	21/28 (75%)		No breakthrough infection	

	mRNA-Exclusive Vaccination Group: 56 patients	Anti-spike IgG quantified by ECLIA (Roche), Architect (Abbott), or Diasorin. Expressed in Binding Antibody Units (BAU)/ml.	None had a history of COVID-19 infection	Detection of anti-spike IgG above the lab's positivity threshold after vaccination.	38/56 (67.78%)	Not reported	No breakthrou gh infection	
Mazzola 2022, France.	143 patients	Chemilumines cent microparticle immunoassay (CMIA, Abbott) Detected anti-spike IgG (RBD of S1 domain)	All 133 analyzed patients were seronegative at baseline 8/143 had prior COVID-19 (excluded from primary analysis)	Anti-spike antibody titers above 50.0 AU/mL (or 7.1 BAU/mL), as per the manufacturer's recommendation for the assay.	38/133 (28.6%)	Not reported	4 severe breakthrou gh infections, 1 death	133 SOT recipients completed

Mrak 2022, Austria	mRNA group: 24 patients	Elecsys Anti-SARS-CoV-2 S immunoassay	No prior SARS-CoV-2 infection	An anti-RBD antibody concentration of over 0.8 Binding Antibody Units per milliliter (BAU/ml).	15/24 (63%)	Not reported	N/A	Median anti-RBD antibody levels were significantly higher in the mRNA-vaccinated group compared to the vector-vaccinated group ($p = 0.004$).
	Vector group: 22 patients		No history of COVID-19		4/22 (18%)			
Narasimhan 2021, USA	Pfizer-BioNTech Group: 48 patients.	IgGSP: Abbott Alinity i	IgGNC testing confirmed one prior	Positive anti-spike IgG response, defined as ≥50 AU/mL	9/48 (19%)	Not reported	N/A	

	Moderna Group: 25 patients.	IgMSP: Abbott Alinity i<br>IgGNC: Abbott Alinity i<br>T-cell response: ImmuKnow® Cylex assay	infection in the LT cohort; others were uninfected.	using the Abbott Alinity i SARS-CoV-2 IgG II assay.	9/25 (36%)	Not reported	N/A	
Osmanodja 2022, Germany	5th dose group, 40 patients	Anti-SARS-CoV-2 ELISA (IgG against S1 domain, EUROIMMUN)<br><br>Electrochemiluminescence immunoassay (ECLIA, Elecsys Anti-SARS-CoV-2, Roche)	No prior covid 19 infection	Positive test result for anti-SARS-CoV-2 spike protein antibodies, measured at least 14 days after a vaccine dose	23 / 40 (57.5%)	N/A	N/A	

Ou 2021, USA	609 patients	Anti-S1 domain of spike protein (EUROIMMUN) Anti-receptor-binding domain (RBD) of spike protein (Roche Elecsys)	No prior COVID-19 diagnosis	Defined as a positive antibody test result (≥1.1 AU for anti-S1 or ≥0.8 U/mL for anti-RBD) after vaccination.	191/400 (47.8%)	Not reported	N/A	There were 400 patients for D2 analysis,
Peled 2021 A, Israel	77 patients	Anti-RBD IgG: "In-house" enzyme-linked immunosorbent assay (ELISA). SARS-CoV-2 pseudo-virus (psSARS-2) neutralization assay.	Not explicitly stated for the cohort.	Defined as having detectable IgG antibodies against the SARS-CoV-2 receptor-binding domain (RBD) following vaccination.	14/77 (18%).	Not provided	Not reported.	

Peled 2021 B, Israel	96 patients	IgG anti-RBD (quantitative ELISA) + pseudovirus neutralization assay	Not explicitly stated for the cohort.<br>No prior infection	Presence of either IgG anti-receptor-binding domain (RBD) antibodies or neutralizing antibodies	64/96 (67%)	Not reported	Not reported	
Perrier 2022, France	825 SOT recipients; Kidney group: 516 patients.	Multiple commercial immunoassays were used (Wantai ELISA, VIDAS [Biomérieux], Alinity i [Abbott], Elecsys [Roche], Atellica [Siemens])	All patients were seronegative	Defined as having a detectable anti-spike antibody level, with a level >260 BAU/mL classified as a strong humoral response.	30/53 (56.6%)	Not reported	Breakthrough infections not reported	
	Liver: 361 patients.				67/70 (95.7%)			

	Heart: 108 patients.				57/77 (74.0%)			
	Lung: 98 patients.				25/35 (71.4%)			
Quiroga 2022, Spain.	BNT162b2: 54 patients	Quantitative chemiluminescence immunoassay (CLIA) — COVID-19 Spike Quantitative Virclia® IgG Monotest (Vircell S.L.)	Previous COVID-19 Infection: 6% (17/283) Baseline Anti-Spike Antibody Positive: 30% (13/43 in humoral response subgroup)	Anti-SARS-CoV-2 Spike IgG antibodies (with a titer >36 IU/mL) 28 days after receiving the complete vaccination schedule. .	6/10 (60%)	Not reported	After second dose: 1 breakthrough infection (vaccine type not specified)	KT recipients had significantly lower anti-Spike antibody titres compared to other CKD groups.<br><br>Previous COVID-19 infection was also associated with higher rates of reactions after the second dose (65% versus 53%)

	mRNA-1273: 225 patients.				14/17 (82%)	Not reported	Not reported	mRNA-1273 is associated with higher titres than BNT162b2.
Rabinowich 2021, Israel.	80 patients	LIAISON SARS-CoV-2 S1/S2 IgG chemiluminescent assay (DiaSorin).<br><br>Architect i2000SR analyzer (Abbott).	No prior Covid 19 infection	Blood test result for SARS-CoV-2 S1/S2 IgG antibodies was >15.0 AU/ml.	38/80 (47.5%)	Not reported	Breakthrough infection not reported	
Rahav 2021, Israel	Kidney Transplant: 111 patients,	Enzyme-linked immunosorbent assay (ELISA) SARS-CoV-2 pseudo-virus neutralization assay	No prior Covid 19 infection	Defined as a positive result for SARS-CoV-2 anti-RBD IgG antibodies with a titer ≥ 1.1. .	50/111 (45.0%)	1.00 (0.80 – 1.24) RBD-IgG GMT (95% CI)	Breakthrough infection not reported	
	Heart Transplant: 80 patients				15/80 (18.8%)	0.55 (0.44 0.68) RBD-IgG GMT (95% CI)		

	Liver Transplant: 36 patients				25/36 (69.4%)	2.14 (1.46 – 3.14) RBD-IgG GMT (95% CI)		
Ruether 2022, Germany	138 patients	DiaSorin LIAISON (anti-S trimer) Roche Elecsys (anti-S RBD) LIAISON	Prior history of COVID-19 was excluded	Defined as an anti-SARS-CoV-2 antibody titer ≥33.8 BAU/mL on the DiaSorin Trimer assay or ≥0.8 U/mL on the Roche RBD assay.	87/ 138 (63%)	Not reported	N/A	

Russo 2021, Italy	82 patients	LIAISON SARS-CoV-2 S1/S2 IgG chemiluminescent assay (DiaSorin). Negative: <12.0 AU/mL.	No prior Covid 19 infection	Detection of anti-spike SARS-CoV-2 antibodies with results ≥12.0 AU/mL	43/82 (52.4%)	Not reported	1 case (1.2%) of mild COVID-19 after the first dose.	
Sanders 2022, The Netherlands	288 patients	Multiplex Immunoassay<br><br>Plaque Reduction Neutralization Test ($PRNT_{50}$)	No prior Covid 19 infection	A SARS-CoV-2 Spike S1-specific IgG antibody concentration of ≥10 BAU/mL.	164/288 (56.9%)	Not reported	Breakthrough infection not reported	

Schmidt 2021, Germany.	40 patients	.IgG ELISA: SARS-CoV-2-QuantiVac (Euroimmun)<br><br>Neutralization Assay: SARS-CoV-2-NeutralISA (Euroimmun)<br><br>T-cell Assay: Flow cytometry (IFNγ, CD69, IL-2, TNFα)	All patients were seronegative	IgG antibody levels ≥35.2 Binding Antibody Units (BAU)/ml against the SARS-CoV-2 spike protein receptor-binding domain.<br>.	12/34 (35.3%)	Not reported	Not reported	
Schramm 2021, Germany.	50 patients	Abbott SARS-CoV-2 IgG II Quant (anti-RBD), Roche Elecsys Anti-SARS-CoV-2 S (anti-RBD), Euroimmun Anti-SARS-CoV-2 ELISA (anti-S1).	All patients were seronegative	Having anti-SARS-CoV-2 IgG antibody titers above the specific cut-off (, ≥7.1 BAU/ml for Abbott) after vaccination	5/50 (10%)	Not reported	Breakthrough infection not reported	

Schrezenmeier 2021, Germany.	Heterologous ChAdOx1 Group: 11 patients	ELISA (Euroimmun) for Anti-S1 IgG/IgA. Surrogate SARS-CoV-2 neutralization test (GenScript).	No prior Covid 19 infection	Developing a positive anti–S1 IgG result (OD ratio ≥1.1) after vaccination.	5/ 11 (45%).	Not reported	1 patient developed severe COVID-19	
	Homologous BNT162b2: 14 patients				.4/ 14 (28%).	Not reported		
Schrezenmeier 2022, Germany.	29 patients				22/29 (76%)	Not reported	No breakthrough infections	
Schwaighofer 2021, Austria.	Homologous mRNA Group: 99 patients,	Elecsys Anti-SARS-CoV-2 S (Roche).<br><br>Surrogate Virus Neutralization Test (sVNT, GenScript).<br><br>QuantiFERON SARS-CoV-2 IGRA (Qiagen).	No Prior Infection	Defined as spike antibody level >0.8 U/mL at 4 weeks.	35/99 (35%)	Not reported	No breakthrough infections	
	Heterologous Vector Group: 98 patients,				42% (41/98)	Not reported		

Seija 2022, Uruguay.	Inactivated Virus (CoronaVac) : 245 patients	COVID-19 IgG QUANT ELISA Kit	No prior confirmed COVID-19.	Presence of specific immunoglobulin G (IgG) antibodies against the receptor-binding domain (RBD) of the SARS-CoV-2 Spike protein.	66/245 (27.8%)	Not reported	No breakthrough infections	
	BNT162b2: 39 patients				36.5% (14 out of 39 patients)	Not reported		
Slizien 2021, Poland	mRNA-1273 Group: 37 patients	LIAISON® SARS-CoV-2 Trimeric-S IgG chemiluminescent immunoassay (Diasorin)	COVID-19 naïve	As an anti-spike IgG antibody titer >12 AU/mL measured 14-21 days after the second vaccine dose. .	23/37 (62.16%)	Not reported	N/A	Independent predictors of higher S-antibody titer among responders were younger age, treatment with no more than two immunosuppressants, and the mRNA-1273 vaccination.
	BNT162b2 Group:105 patients				50/105 (47.6%)	Not reported	N/A	

Hod B 2023, Israel	447 patients (Humoral response subgroup = 74),	SARS-CoV-2 IgG II Quant and A SARS-CoV-2 pseudovirus (psSARS-2) neutralization assay.	Patients were seronegative	Positive response was defined as the presence of NAs capable of reducing viral replication by at least 50% at a ≥16-fold dilution	70/74 (94.6%)	Not reported	71 (49.3%) breakthrough infections	
Thomson 2022, United Kingdom	ChAdOx1(V 1/2) - mRNA-1273(V3). 31 patients	Abbott Architect SARS-CoV-2 IgG Quant II CMIA (measured anti-Spike IgG antibodies). T-SPOT® Discovery SARS-CoV-2	Covid 19 naive	Defined as anti-Spike IgG ≥7.1 BAU/mL using the Abbott Architect SARS-CoV-2 IgG Quant II CMIA assay.	15/31 (48.4%)	Not reported	N/A	
	ChAdOx1(V 1/2) - BNT162b2(V3),245 patients ,				181/245 (73.9%)	Not reported	N/A	

	BNT162b2(V1/2) - mRNA-1273(V3), 25 patients	(measured IFN-γ producing T-cells).			18/25 (72%)	Not reported	N/A	
	BNT162b2(V1/2/3) (Homologous), 285 patients				231/285 (81.1%)			
	BNT162b2(V1-4) (Fully Homologous), 115 patients,				86.1% (99/115)			
	ChAdOx1(V1/2) - BNT162b2(V3/4), 89 patients				82.0% (73/89)			

Thuluvath 2021, USA	62 patients	Roche Elecsys® Anti-SARS-CoV-2 S (semi-quantitative) for antibodies to the spike protein	Covid 19 naïve	Antibody titer of >250 U/ml	24/62 (38.7%)	Not reported	Breakthrough infections not reported	
Timmermann 2021, Germany	118 patients	Anti-SARS-CoV-2-Elisa (Euroimmun) and Elecsys Anti-SARS-CoV-2 assay (Roche)	All patients were seronegative	Detectable IgG antibodies	92/118 (78.0%)	Not reported	No breakthrough infections	
Tsoutsoura 2023, Greece.	144 patients	Chemiluminescent microparticle immunoassay (CMIA) for Anti-RBD IgG (Abbott SARS-CoV-2 IgG II Quant)	All patients were seronegative	An antibody level of ≥50 AU/mL	93/134 (69.4%)	Not reported	Not reported	
Tylicki 2022, Poland	BNT162b2 Group: 60 patients	LIAISON® SARS-CoV-2	Some patients had	Seroconversion was defined as an	40/60 (66.67%)	Not reported	Not reported	

	Moderna: 23 patients	TrimericS IgG test (DiaSorin)	prior infection	anti-S IgG titer >33.8 BAU/mL	19/23 (82.61%)			
Vaiciuniene 2021, Lithuania	136 patients	QuantiVac ELISA (Euroimmun) for anti-SARS-CoV-2 Spike IgG	All patients were SARS-CoV-2 naïve	Result ≥ 35.2 BAU/mL	39/136 (28.7%)	Not reported	N/A	
Wijtvliet 2022, Belgium	mRNA-1273 Group in KTR: 42 patients,	In-house Luminex assay for anti-Receptor-Binding Domain (RBD) IgG antibodies.	No prior infection	Anti-RBD IgG positivity (signal-to-noise ratio >1) after vaccination.	32/42 (76.2%)	Geometric Mean Anti-RBD IgG: 7.27 (SD 2.63)	N/A	
	BNT162b2 Group in KTRs: 91 patients				51/91 (56.0%)	Geometric Mean Anti-RBD IgG: 5.37 (SD 2.73)		

Yi 2021, USA	145 patients	Anti-SARS-CoV-2 total antibody, anti-SARS-CoV-2 IgG, anti-spike IgG titer, anti-nucleocapsid IgG	No prior infection	As developing anti-SARS-CoV-2 total antibody, IgG, or anti-spike IgG after the first vaccine dose.	8/145 (5.5%)	Not reported	Not reported	
Zadok 2021, Israel	42 patients	Abbott Architect SARS-CoV-2 IgG II Quant assay (anti-spike IgG)	No prior infection	An anti-spike IgG (S-IgG) antibody titer of ≥50 AU/mL was interpreted as seropositive.	18/37 (49%)	GMT 426 AU/mL (IQR 106-884)	Breakthrough infections	A booster (second) dose was beneficial, converting 36% of initial non-responders into seropositive individuals.
Rozen-Zvi 2021, Israel	308 patients	SARS-CoV-2 IgG II Quant (Abbott) for anti-spike (anti-S) antibodies.	Not specified for the entire cohort	An anti-spike IgG antibody level ≥50 AU/mL measured 2–4 weeks after the second vaccine dose.	112/308 (36.4%)	Not reported	4 (All in the seronegative group)	

Midtvedt 2021, Norway	141 patients,	Multiplexed bead-based flow cytometric assay.<br><br>Bead-based array.	No known history of COVID-19	A threshold for positive response set at ≥1.0 Binding Antibody Unit per milliliter (BAU/mL).	25/141 (17.7%)	Not reported	N/A	
Midtvedt 2022, Norway	188 patients	Binding antibody assay (measuring anti-RBD IgG in BAU/ml) and a functional neutralizing antibody assay based on a bead-based ACE2-RBD interaction test.	No known history of COVID-19	Anti-SARS-CoV-2 IgG antibody level above 200 BAU/ml	79/188 (42%)	Not reported	N/A	

Miele 2021, Italy.	16 patients	Anti-Spike protein IgG detected using LIAISON SARS-CoV-2 S1/S2-IgG chemiluminescent assay (DiaSorin).	Not stated	Anti-SARS-CoV-2 S1/S2 IgG concentration >15 AU/ml	6/16 (37%)	Not reported	N/A	
Ducloux, 2021, France	153 patients	SARS-CoV-2 immunoassay (Abbott®) detecting IgG antibodies to the RBD of the spike protein.	COVID-19 naïve	≥50 UA/mL	81/153 (53%)	N/A	N/A	
Erol 2021, Turkey	Sinovac subgroup: 31 patients	SARS-CoV-2 IgG II Quant assay (Abbott) for anti-spike IgG antibodies.	COVID-19 naïve	Seropositivity defined as IgG ≥ 50 AU/mL	21/31 (67.7%)	Not reported	Not reported	
	BioNTech group: 17 patients				14/17 (82.4%)			

Haidar 2022 USA	183 patients	Beckman Coulter SARS-CoV-2 Spike RBD IgG (semi-quantitative) Pseudovirus neutralization assay (subgroup)	Excluded if prior COVID-19 infection	Reactive SARS-CoV-2 Spike protein IgG (≥1.00 signal/cutoff)	138/450 (30.7%)	Not reported	Not reported	
Middleton 2021, United Kingdom	698 patients	Siemens immunoassay for spike protein S1 receptor-binding domain (RBD**)**	Prior to vaccination: 55/298 (18.4%) had detectable antibodies (35 asymptomatic).	Development of detectable COVID-19 antibodies following first vaccine dose	16/70 (22.9%)	Not reported	Not reported	
Mulder 2022. The Netherlands	476 patients	Liaison SARS-CoV-2 Trimeric IgG assay (DiaSorin, Italy)	All patients were seronegative	Detectable IgG SARS-CoV-2 anti-spike antibodies	376/476 (79.0%)	Not reported	Not reported	
Nazaruk 2021, Poland	61 KTRs	SARS-CoV-2 IgG II Quant	KTRs: 8.2% (5/61) PCR-	Anti-SARS-CoV-2 spike protein	28/ 49 (57.1%).	Not reported	Not reported	

	55 LTRs	test (Abbott; CMIA) for anti-S1 antibodies (AU/mL).	confirmed prior infection.<br><br>LTRs: 9.1% (5/55) PCR-confirmed prior infection.	IgG antibody (anti-S1 Ab) titer > 50 AU/mL	40/ 45 (88.9%).			
Correia 2022, Portugal	70 patients	Alinity i SARS-CoV-2 IgG II chemiluminescent immunoassay (CMIA), Abbott	No prior Covid 19 infection	Anti-spike IgG titer ≥ 7.1 BAU/mL (≥50 U/mL)	51/70 (67%)	Not reported	Not reported	
	61 patients				25/61 (33%)			
Prendecki 2021, United Kingdom	Previous infection: 152 patients.	SARS-CoV-2 IgG and IgG Quant II (Abbott) for anti-spike (anti-S) and anti-	Previous infection group: 152 patients	Detectable anti-S antibodies. Cutoff for positive result: >7.1 BAU/mL.	144/152 (95%)	Not reported	Not reported	
	Infection naïve patients, BNT162b2 group: 410 patients.		No prior Covid infection		269/410 (66%)			

	Infection naïve patients, ChAdOx1 group: 358 patients.	nucleocapsid antibodies.	No prior Covid infection.		156/358 (44%)	Not reported	Not reported	
Saharia, 2022, United States	53 patients	ELISA (for antibody titers). cPass surrogate virus neutralization test (sVNT)	No prior Covid	Good Responder (GR) >90% inhibition; Reduced Responder (RR) 30-89%	18/32 (56.3%)	Not reported	Not reported	
Sakai 2022, Japan	56 patients	SARS-CoV-2 S-IgG (IC) Assay Reagent (Fujirebio Inc.), measuring IgG to the S receptor-binding domain (RBD).	No prior infection	RBD-IgG titer >1.0 AU/mL.	44/56 (78.6%)	Not reported	Not reported	

Spinner 2022, United States	40 patients	SARS-CoV-2 Anti–Spike IgG quantitated using the FDA-EUA approved Vitros 5600 immunoassay.	7/40 post-HTx patients had known pre-vaccination SARS-CoV-2 infection	Detectable SARS-CoV-2 Anti–Spike IgG antibody (>1.0 AU/mL).	28/40 (70%)	Not reported	3 patients (3.1% of total vaccinated) had symptomatic breakthrough COVID-19	
Strauss 2021, USA	161 patients	Roche Elecsys (anti-RBD) EUROIMMUN (anti-S1)	No prior Covid 19	Antibody-positive per manufacturer cutoffs: Roche: ≥0.80 U/mL EUROIMMUN: ≥1.1 arbitrary units	130/161 (81%)	Not reported	Not reported	
Toniutto, 2022, Italy	Covid 19 naïve patients: 131 patients.	Roche Elecsys Anti-SARS-CoV-2 S (targeting	12 patients had prior infection	Anti-SARS-CoV-2 s-RBD antibody titer ≥ 0.8 U/ml.	97/123 (78.8%)	Not reported	Not reported	

	Covid 19 recovered patients: 12 patients.	receptor-binding domain, RBD).<br><br>iFlash–Shenzhen Yhlo Biotech Co. Ltd (used to define baseline COVID-19 status).			12./12 (100%)			
Yanis 2021, USA	56 patients	ELISA (anti-RBD, anti-ECD IgG), Luminex (anti-RBD IgG), antigen-induced marker assay (CD4+/CD8+ T-cell responses)	No prior infection	Anti-ECD IgG level ≥ 1.07 EU	11/51 (21.6%)	Not reported	Not reported	

CLIA: Chemiluminescence Immunoassay; ELISA: Enzyme-Linked Immunosorbent Assay; ECLIA: Electrochemiluminescence Immunoassay; sVNT: Surrogate Virus Neutralization Test; PRNT: Plaque Reduction Neutralization Test; IGRA: Interferon-Gamma Release Assay; ELISpot: Enzyme-Linked Immunospot (Assay); FACS: Fluorescence-Activated Cell Sorting; CITE-seq: Cellular Indexing of Transcriptomes and Epitopes by Sequencing; scRNA-seq: Single-Cell RNA Sequencing; CMIA: Chemiluminescent Microparticle Immunoassay; RBD: Receptor-Binding Domain (of the SARS-CoV-2 spike protein); GMT: Geometric Mean Titer; CI:

Confidence Interval; IQR: Interquartile Range; SD: Standard Deviation; SEM: Standard Error of the Mean; AU/mL: Arbitrary Units per Milliliter; BAU/mL: Binding Antibody Units per Milliliter; OD: Optical Density; S/CO: Signal-to-Cut-Off Ratio; ID_{50}: 50% Inhibitory Dose; ACE2: Angiotensin-Converting Enzyme 2; MFI: Mean Fluorescence Intensity; RT-PCR: Reverse Transcription Polymerase Chain Reaction.

Electronic Supplementary Table 4. Leave-one-out sensitivity analysis.

Group Type	Group Name	Most Influential Study	Maximum Difference	Maximum Percent Change
Vaccine Type	BNT162b2	Perrier	-1.0	-2.0
	CoronaVac	Erol	-9.0	-31.4
	mRNA-1273	Benotmane	2.6	4.7
Organ	Liver	Perrier	-2.9	-3.7
	Kidney	Hod	-1.2	-2.6
	Mixed	Schramm	1.9	4.3
	Lung	Havlin	13.6	46.7
	Heart	Peled	6.8	14.8
Dose	2	Danthu	0.8	1.8
	1	Kho	-16.4	-59.7
	3	Chavarot	3.5	5.8
	4	Perrier	-4.1	-4.9
Prior COVID	No	Danthu	0.8	1.7
	Yes	Guarino	7.7	8.6

Electronic Supplementary Table 5. Safety of COVID-19 vaccines amongst the included studies.

Study (Author, Year)	Population Analyzed (N)	Any Local AE n/N	Any Systemic AE n/N	Serious Adverse Events (SAEs) n/N (%)	SAEs Possibly Related to Vaccine (always unrelated) - if its possibly related.	Key Findings / Notes
Azzi 2021, USA	No prior Covid group: 76 patients	Not reported	Not reported	Not reported	Not reported	
	Prior Covid group: 21 patients	Not reported	Not reported	Not reported	Not reported	
Cholankeril, 2022, USA	69 patients	Not reported	Not reported	Not reported	Not reported	
Westhoff 2021, Germany	10 patients	Not reported	Not reported	Not reported	Not reported	
D'Offizi, 2021, Italy	61 patients	Not reported	Not reported	Not reported	Not reported	

Sadioglu, 2021, Turkey	85 patients	Inoculation site pain: 2/85 (2.3%)	Arthralgia, myalgia, fatigue: 6/85 (7%); Headache: 2/85 (2.3%); Fever: 1/85 (1.1%); Dizziness: 2/85 (2.3%); Sore throat: 1/85 (1.1%); Chest pain: 1/85 (1.1%); Hypertension: 1/85 (1.1%)	Not reported	Not reported	
Sattler 2021, Germany	39 patients	Not reported	Not reported	Not reported	Not reported	
Davidov, 2021, Israel	76 patients	Not in detail	Not in detail	Not in detail	Not in detail	
Rashidi-Alavijeh 2021,Germany	43 patients	Not reported	Not reported	Not reported	Not reported	No episodes of acute organ rejection
Rincon-Arevalo 2021, Germany	40 patients	Not reported	Not reported	Not reported	Not reported	Safety was not assessed

Balsby 2022, Denmark	358 patients	Not reported	Not reported	Not reported	Not reported	Liver disease and diabetes = weak response; liver transplants responded best, lung worst.
Bergman 2021, Sweden	83 SOT	Exact number not given.	Exact number not given.	Exact number not given.	Exact number not given.	Local side effects in SOT recipients were similar to controls, while systemic effects were likely comparabl e or lower.
Benotmane 2021 A, France	204 patients	Not reported	Not reported	Not reported	Not reported	Safety not assessed

Marion, 2021, France	Kidney: 271patients	Not reported	Not reported	One kidney transplant recipient had a 50% increase in serum creatinine related to drug-induced dehydration	Not detailed	
	Liver: 58 patients	Not reported	Not reported	One liver transplant recipient developed paraesthesia of the lower limb.		
	Thoracic organs: 12 patients	Not reported	Not reported	Not reported		
	Pancreas: 5 patients	Not reported	Not reported	Not reported		

Del Bello 2022, France	396 patients	Not reported	Not reported	Not reported	Not reported	The third dose was found to be safe, with no serious adverse events or acute organ rejection episodes observed.
Benotmane 2021 B, France	242 patients	Not reported	Not reported	Not reported	Not reported	Safety not assessed
Benotmane 2021 C. France	159 patients	Not reported	Not reported	Not reported	Not reported	Safety not assessed
Cao 2022, USA	37 patients	Not reported	Not reported	Not reported	Not reported	Safety not assessed

Cotugno 2022, Italy	34 patients	Not reported	Not reported	Not reported	Not reported	The vaccine was safe with no reported cases of graft rejection or serious adverse events.
Devresse 2021, Belgium	90 patients.	Not reported	Not reported	Not reported	Not reported	Safety not assessed
Firket 2021, Belgium	10 patients with prior Covid 19	Not reported	Not reported	Not reported	Not reported	Safety not assessed
	10 patients with prior Covid 19	Not reported	Not reported	Not reported	Not reported	Safety not assessed
Georgery 2021, Belgium.	79 patients	Not reported	Not reported	Not reported	Not reported	Safety not assessed
Masset 2021, France	456 patients	Not reported	Not reported	Not reported	Not reported	Safety not assessed
Griessbach 2022, Switzerland	26 patients	Not reported	Not reported	Not reported	Not reported	Safety not assessed

Havlin 2022, Czech Republic	15 patients	Not reported	Not reported	Not reported	Not reported	The third dose was safe: no systemic adverse events, graft rejection, or lung decline at 3 months.
Holden 2021, Denmark	80 patients	Not reported	Not reported	Not reported	Not reported	Findings show vaccinated SOT recipients remain high-risk

Hoffman 2021, The Netherlands	No COVID-19 before vaccination: 79 patients	Not reported	Not reported	Not reported	Not reported	Authors conclude lung transplant recipients remain at risk for severe COVID-19 despite 2 vaccine doses.
	Had COVID-19 before vaccination: 12 patients	Not reported	Not reported	Not reported	Not reported	
Husain 2021. USA	Moderna group: 12 patients.	Not reported	Not reported	Not reported	Not reported	Vaccinated transplant recipients should continue precaution s, as protection may be limited.
	Pfizer: 16 patients.	Not reported	Not reported	Not reported	Not reported	

Marinaki 2021, Greece	34 patients	Not reported	Not reported	Not reported	Not reported	
Massa 2021,France	61 patients.	Injection-site pain: 41/61 (67.2%)	Fatigue: 13/61 (21.3%)<br>Headache: 7/61 (11.5%)<br>Diarrhea: 7/61 (11.5%)<br>Fever: 4/61 (6.6%)<br>Myalgia: 2/61 (3.3%)<br>Rhinorrhea: 2/61 (3.3%)<br>Nausea and vomiting: 1/61 (1.6%)<br>Hypertension: 1/61 (1.6%)<br>Anorexia: 1/61 (1.6%)<br>Vertigo: 1/61 (1.6%)<br>Rash: 1/61 (1.6%)	Not detailed	Not reported	Treatment with antiprolifer ative drugs (e.g., mycophen olate) was the strongest predictor of failing to seroconver t even after three doses.
Chavarot 2021, France A	`101 patients	Not reported	Not reported	Not reported	Not reported	Safety not assessed

Chavarot 2021, France B	12 (from 181 group) (safety subgroup)	Not reported	Not reported	Hospitalization: 8/12 (67%) ICU admission: 3/12 (25%) Deaths: 6/12 (50%)	Not reported	Hospitalization rate highlighted the lack of efficacy of the vaccine in this group
Noble 2021, France	57 patients	Not reported	Not reported	Not reported	Not reported	
Pedersen 2021, Denmark	58 patients	Not reported	Not reported	Not reported	Not reported	
Medina-Pestana 2022, Brazil.	3354 patients	Local pain or tenderness: 378/3354 (11%)	Headache: 178/3354 (5%) Myalgia: 160/3354 (5%) Runny nose: 113/3354 (3%)<br><br>Diarrhea: 93/3354 (3%) Sore throat: 65/3354 (2%) Fever: 39/3354 (1%)	44 (72%) required hospitalization and 16 (26%) died 14–49 d after the first vaccine dose.	One episode of acute cellular rejection (Banff IB) 6 days post-vaccination in a nonadherent patient (0.03%)	

Shostak 2021, Israel	168 patients	Localized tenderness at injection site 108/168 (64.3%)	Fatigue 32/168 (19%)	Not reported	Not reported	The vaccine was found to be safe. No serious adverse events, vaccine-related deaths, or episodes of acute transplant rejection were reported.
Stumpf 2021 A, Germany	71 patients	Not reported	Not reported	Not reported	Not reported	Safety not assessed
Stumpf 2021 B, Germany.	368 patients (99 patients for BNT162b2)	Arm pain: 78/376 (20.7%)	Joint pain: 10/376 (2.7%)	Severe allergic	Not reported	

	368 patients (234 for mRNA-1273)		Fever: 14/376 (3.7%)<br><br>Shivering: 6/376 (1.6%)	reaction: 0/376 (0%) Hospitalization due to vaccination: 6/376 (1.6%)	Not reported	Adverse effects were overall reported as mild and less frequent in dialysis patients compared to the MP and KTR cohorts.
Villanego 2021, Spain	91 patients	Not reported	Not reported	5 hospitalizations (33.3% of breakthrough cases), 1 death.	Not reported	Infection rate: 1.8% in transplant recipients vs 0.01% in general population
Benning 2022, Germany.	40 patients	Not reported	Not reported	Not reported	Not reported	
	7 patients	Not reported	Not reported	Not reported	Not reported	
	2 patients	Not reported	Not reported	Not reported	Not reported	

Boyarsky 2021 A, United States	Janssen vaccine group 12 participants.	Not reported	Not reported	Not reported	Not reported	
	mRNA vaccine group: 725 participants	Not reported	Not reported	Not reported	Not reported	
Boyarsky, 2021 B, USA	436 patients	Not reported	Not reported	Not reported	Not reported	
Boyarsky, 2021 C USA	658 patients	Not reported	Not reported	Not reported	Not reported	
Brandstetter 2022, Austria	324 patients	Mild symptoms. Specifics not given	Mild symptoms. Specifics not given	2/ 324 of biopsy-proven acute rejection (BPAR) (0.62%)	1 graft rejection vaccine related	No severe adverse events, but 2 graft rejections.

Bruminhent 2022, Thailand	mRNA vaccine: 40 patients	Not detailed	Muscle aches: 8/40 (20%)<br>Fever: 6/ 40 (15%)<br>Sleepiness: 3/40 (8%)	1 transient hemiparesis case	Not reported	Safety: Mild AEs; no graft rejections or severe events
	Viral vector vaccine group (V group): 37 patients	Not detailed	Muscle aches: 7/37 (19%)<br>Fever: 5/37 (14%)<br>Sleepiness: 6/37 (16%)<br>Increased appetite: 3/37 (8%)	1 transient hemiparesis case	Not reported	Safety: Mild AEs; no graft rejections or severe events
Buchwinkler 2021, Austria	216 patients	Not reported	Not reported	Not reported	Not reported	
Chukwu 2022, United Kingdom	373 patients (total), BNT162b2: 216 patient s	Not reported	Not reported	Deaths: 3 (all in non-responder group)<br>No deaths in seropositive group	Not reported	

	373 patient (total), AZD1222: 84 patients	Not reported	Not reported	Deaths: 3 (all in non-responder group)<br>No deaths in seropositive group	Not reported	
Crane 2021, USA	25 patients	Not reported	Not reported	Not reported	Not reported	Findings support vaccinatin g adolescent KTRs and exploring boosters or immunosu ppression changes.
Crespo 2021, Spain	90 patients	Not reported	Not specified for transplant patients	Not specified for transplant patients	Not reported	

Cucchiari 2021, Spain	148 patients,	Injection site pain: 111/148 (75.0%)	Fatigue: 40/148 (27%)	Not reported	Not reported	mRNA-1273 vaccine is safe in kidney transplant recipients.
Danthu 2021, France	74 patients	Not reported	Not reported	Not reported	Not reported	
Dov 2022, Israel	252.patients	Not reported	Not reported	Not reported	Not reported	
Fernández-Ruiz 2021, Spain	44 patients	Pain at injection site: 6/44 (13.64%)	Headache: 3/44 (6.82%) Fatigue: 2/44 (4.5%) Fever: 1/44 (2.27%) Tachycardia: 1/44 (2.27%) Nausea: 1/44 (2.27%)	Not reported	Not reported	
Frolke 2022, The Netherlands.	2092 patients	Not reported	Not reported	Not reported	Not reported	
	1401 patients	Not reported	Not reported	Not reported	Not reported	
Grupper 2021, Israel	136 patients	Local pain: 84 /161 (52.2%)	Not reported	Not reported	Not reported	

Guarino 2022, Italy	No Prior COVID: 444 patients	Pain: 278/444 (62.6%)<br>Redness: 29/444 (6.5%)	Swelling: 23/444 (5.2%)<br>Fatigue: 142/444 (32.0%)<br>Headache: 181/444 (40.8%)<br>Myalgia: 94/444 (21.17%)<br>Fever/Chills: 64/444 (14.4%)<br>Nausea/vomiting: 1/444 (0.23%)<br>Diarrhea: 3/444 (0.68%)	Not reported	Not reported	

	Previous COVID-19: 48 patients	Pain: 47.9% (23/48)<br><br>Redness: 12.5% (6/48)	Swelling: 8.3% (4/48)<br><br>Fatigue: 39.6% (19/48)<br><br>Headache: 54.2% (26/48)<br><br>Fever/Chills: 14.6% (7/48)<br>Myalgia: 11/48 (22.9%)<br>Diarrhea: 1/48 (2%)	Not reported	Not reported	
Hall 2021 A, Canada	127 patients	Not reported	Not reported	Not reported	Not reported	
Hall 2021 B, Canada	60 patients	Not reported	Not reported	Not reported	Not reported	
Hallet 2021, USA	Heart transplant recipient: 134 patients	Pain at injection site: 180/237 (76%)	Fatigue: 133/237 (56.12%)<br><br>Headache: 92/237 (39%)	Not reported	Not reported	
	Lung Transplant (LT) Recipients: 103 patients			Not reported	Not reported	
Hamm 2022,Denmark	200 patients	Not reported	Not reported	Not reported	Not reported	

Harberts 2022, Germany	106 patients	Not reported	Not reported	Not reported	Not reported	
Haskin 2021, Israel	38 patients	Injection site pain: 24/37 (65%)<br><br>Redness: 6/37 (16%)	Fatigue: 15/37 (41%)<br><br>Headache: 13/37 (35%)<br><br>Muscle pain: 12/37 (32%)	Not reported	Not reported	
Havlin 2021, Czech Republic	LTRs patients' post-COVID: 33 patients	Not reported	Not reported	Not reported	Not reported	
	Vaccinated LTRs: 48 patients	Not reported	Not reported	One episode of acute cellular rejection after the 1st dose (reason for not receiving 2nd dose). 1/48 (2.1%)	Not reported	
Heinzel 2022, Austria	Homologous (mRNA) Group: 85 patients	Not reported	Not reported	Not reported	Not reported	

	Heterologous (Vector) Group: 84 patients	Not reported	Not reported	Not reported	Not reported	
Herrera 2021, Spain	Liver Transplant Recipients: 58 patients	Pain at injection site: 83/104 (80%)	Fatigue: 16/104 (15%)<br><br>Swelling: 12/104 (12%)<br><br>Low-grade fever: 7/104 (7%)	Not reported	Not reported	
	Heart Transplant Recipients: 46 patients			Not reported	Not reported	
Hod A 2021, Israel	120 patients	Pain at injection site: 12/112 (10.4%)	Fatigue: 11/112 (9.8%)<br>Headache: 6/112 (5.4%)<br>Myalgia: 4/112 (3.6%)	Not reported	Not reported	
Kantauskaite 2021, Germany	225 patients	Not reported	Not reported	Not reported	Not reported	
Karaba 2022, United States	25 patients	Not reported	Not reported	Not reported	Not reported	

Kho 2022, The Netherlands.	Group 1A: Single Dose mRNA-1273 (Control Group), 71 patients randomized	Pain at injection site: 64/71 (90%)<br><br>Induration: 8/71 (11%)<br><br>Erythema: 5/71 (7%)	Fatigue: 36/71 (51%)<br><br>Headache: 25/71 (35%)<br><br>Myalgia: 32/71 (45%)<br><br>Chills: 15/71 (21%)<br><br>Arthralgia: 21/71 (30%)<br><br>Nausea: 13/71 (18%)<br><br>Fever: 2/71 (3%)	Not reported	Not reported	

	Group 1B: Double Dose mRNA-1273, 73 patients randomized	Pain at injection site: 67/73 (92%)<br><br>Induration: 17/73 (23%)<br><br>Erythema: 10/73 (14%)	Fatigue: 44/73 (60%)<br><br>Headache: 31/73 (42%)<br><br>Myalgia: 43/73 (59%)<br><br>Chills: 27/73 (37%) -<br><br>Arthralgia: 19/73 (26%)<br><br>Nausea: 16/73 (22%)<br><br>Fever: 5/73 (7%)	Not reported	Not reported	

	Group 1C: Heterologous Ad26.COV2-S: 76 randomized	Pain at injection site: 38/76 (50%)<br><br>Induration: 5/76 (7%)<br><br>Erythema: 3/76 (4%)	Headache: 40/76 (53%)<br><br>Fatigue: 37/76 (49%)<br><br>Myalgia: 31/76 (41%)<br><br>Arthralgia: 24/76 (32%)<br><br>Chills: 13/76 (17%)<br><br>Nausea: 12/76 (16%)<br><br>Fever: 1/76 (1%)	Not reported	Not reported	
Korth 2021, Germany	23 patients	Not reported	Not reported	Not reported	Not reported	
Kumar 2022, Canada	60 patients	Not reported	Not reported	Not reported	Not reported	
Marlet 2021, France.	After 3rd Dose: 160 patients	Not reported	Not reported	Not reported	Not reported	

Masset 2022, Germany	Heterologous Vaccination Group: 28 patients	Not reported	Not reported	Not reported	Not reported	
	mRNA-Exclusive Vaccination Group: 56 patients	Not reported	Not reported	Not reported	Not reported	
Mazzola 2022, France.	143 patients	Injection-site pain: 37/143 (25.7%)	Fatigue: 21/143 (14.2%) Headache: 9/143 (6.3%)	Not reported	Not reported	

Mrak 2022, Austria	mRNA group: 24 patients	Local pain at injection site: 9/24 (38%)	Fatigue: 10/24 (41.67%)<br><br>Headache: 8/24 (33.3%)<br><br>Myalgia: 9/24 (37.5%)<br><br>Arthralgia: 4/24 (16.67%)	Not reported	Not reported	
	Vector group: 22 patients	Local pain at injection site: 5/22 (23%)	Fatigue: 11/22 (50%)<br>Headache: 9/22 (40.9%)<br>Myalgia: 7/22 (31.8%)<br>Arthralgia: 7/22 (31.8%)	Not reported	Not reported	
Narasimhan 2021, USA	Pfizer-BioNTech Group: 48 patients	Not reported	Not reported	Not reported	Not reported	
	Moderna Group: 25 patients	Not reported	Not reported	Not reported	Not reported	
Osmanodja 2022, Germany	5th dose group, 40 patients	Not reported	Not reported	Not reported	Not reported	

Ou 2021, USA	609 patients	Pain: 21% (84/400)<br><br>Swelling: 2.0% (8/400)<br><br>Erythema (Redness): 1.0% (4/400)	Fatigue: 38% (83/400)<br><br>Headache: 21% (36/400)<br><br>Myalgias: 6% (24/400)<br><br>Chills: 11% (44/400)<br><br>Fever: 3% (12/400)<br><br>Diarrhea: 2.0% (8/400)<br><br>Vomiting: 1.0% (4/400)	Not reported	Not reported	
Peled 2021 A, Israel	77 patients	Not reported	Not reported	Not reported	Not reported	
Peled 2021 B, Israel	96 patients	Not reported	Not reported	Not reported	Not reported	

Perrier 2022, France	825 SOT recipients; Kidney group: 516 patients.	Not reported	Not reported	Not reported	Not reported	
	825 SOT recipients; Liver: 361 patients.	Not reported	Not reported	Not reported	Not reported	
	825 SOT recipients; Heart: 108 patients.	Not reported	Not reported	Not reported	Not reported	
	825 SOT recipients; Lung: 98 patients.	Not reported	Not reported	Not reported	Not reported	
Kamar 2021, France	101 patients	Not reported	Not reported	Not reported	Not reported	
Quiroga 2022, Spain.	BNT162b2: 54 patients.	Not reported	Not reported	1/283 myocardial infarction (0.35%). 4 days after first dose of BNT162b2)	Not reported	

	mRNA-1273: 225 patients	Not reported	Not reported	Not reported	Not reported	
Rabinowich 2021, Israel.	80 patients	Exact number not reported	Exact number not reported	Not reported	Not reported	
Rahav 2021, Israel	Kidney Transplant: 111 patients,	Exact number not reported	Exact number not reported	Not reported	Not reported	
	Heart Transplant: 80 patients	Exact number not reported	Exact number not reported	Not reported	Not reported	
	Liver Transplant: 36 patients	Exact number not reported	Exact number not reported	Not reported	Not reported	
Ruether 2022, Germany	138 patients	Pain/Swelling: 82/ 138 (59%) Redness: 3/138 patients (3%)	Fever: 12/ 138 (9%) Fatigue: 51/ 138 (37%) Headache: 31/ 138 (23%) Vomiting: 7/1 38 (5%) Muscle pain: 26/138 (19%) Diarrhea: 7/13 8 (5%)	Not reported	Not reported	

Bertrand 2021 A, France	45 patients	Not reported	Not reported	Not reported	Not reported	
Bertrand 2021 B, France	80 patients	Not reported	Not reported	Not reported	Not reported	
Bertrand 2021 C, France	235 patients.	Not reported	Not reported	Not reported	Not reported	
Russo 2021, Italy	82 patients	Pain in the site of inoculation: 27/82 (32.9%)	Fever: 1/82 (1.2%)<br>Flu-like symptoms: 1/82 (1,2%)	Not reported	Not reported	

Sanders 2022, The Netherlands	288 patients	Erythema: 53/288 (18.4%) Induration: 85/288 (29.5%) Pain at Injection Site: 269/288 (93.4%)	Arthralgia: 99/288 (34.4%) Fatigue: 184/288 (63.9%) Fever: 24/288 (8.3%) Chills: 102/288 (35.4%) Headache: 147/288 (51.0%) Myalgia: 157/288 (54.5%) Nausea: 80/288 (27.8%)	Graft Rejection (unrelated): 1/288 (0.3%) Deaths (unrelated): 1/288 (0.3%)	SAEs Possibly Related to Vaccination: 2/288 (0.69%)<br><br>Cellulitis: 1/288 (0.347%)<br><br>Malaise: 1/288 (0.347%)	
Schmidt 2021, Germany.	40 patients	Not reported	Not reported	Not reported	Not reported	
Schramm 2021, Germany.	50 transplant recipients,	Not reported	Not reported	Not reported	Not reported	
Schrezenmeier 2021, Germany.	Heterologous ChAdOx1 group: 11 patients	Not reported	Not reported	Not reported	Not reported	

	Homologous BNT162b2: 14 patients	Not reported	Not reported	Not reported	Not reported	
Schrezenmeier 2022, Germany.	29 patients	Not reported	Not reported	Not reported	Not reported	
Schwaighofer 2021, Austria.	Homologous mRNA Group: 99 patients	Exact numbers not given	Not detailed	1/99 (1%) urinary tract infection 1/99 (1%) symptomatic hypokalemia.	Not reported	Significant ly more patients in the mRNA vaccine group reported injection site pain
	Heterologous Vector Group: 98 patients	Not reported	Not detailed	1/98 (1%) urinary tract infection 1/98 (1%) myocardial infarction. thrombophleb itis 1/98 (1%)		
Seija 2022, Uruguay.	Inactivated Virus (CoronaVac): 245	Not reported	Not reported	Not reported	Not reported	
	BNT162b2: 39 patients.	Not reported	Not reported	Not reported	Not reported	

Slizien 2021, Poland	mRNA-1273 Group: 37 patients	Not reported	Not reported	Not reported	Not reported	
	BNT162b2 Group: 105 patients	Not reported	Not reported	Not reported	Not reported	

Hod B 2023, Israel	447 patients (Humoral response subgroup = 74),	Pain at injection site: 56/74 (75.7%)<br><br>Swelling: 7/74 (9.4%)<br><br>Redness: 5/74 (6.8%)	Fatigue: 20/74 (27.0%)<br><br>Myalgia (Muscle pain): 13/74 (17.6%)<br><br>Headache: 8/74 (10.8%)<br><br>Chills: 4/74 (5.4%)<br><br>Fever: 3/74 (4.1%)<br><br>Nausea/Vomiting: 2/74 (2.7%)<br><br>Paresthesia (Tingling): 0/74 (0%)	Not reported	Not reported	
Thomson 2022, United Kingdom	ChAdOx1(V1/2) - mRNA-1273(V3). 31 patients	Not reported	Not reported	Not reported	Not reported	

	ChAdOx1(V1/2) - BNT162b2(V3), 245 patients ,	Not reported	Not reported	Not reported	Not reported	
	BNT162b2(V1/2) - mRNA-1273(V3), 25 patients	Not reported	Not reported	Not reported	Not reported	
	BNT162b2(V1/2/3) (Homologous), 285 patients ,	Not reported	Not reported	Not reported	Not reported	
	BNT162b2(V1-4) (Fully Homologous), 115 patients,	Not reported	Not reported	Not reported	Not reported	
	ChAdOx1(V1/2) - BNT162b2(V3/4), 89 patients	Not reported	Not reported	Not reported	Not reported	
Thuluvath 2021, USA	62 patients	Not reported	Not reported	Not reported	Not reported	
Timmermann 2021, Germany	118 patients	Not reported	Not reported	Not reported	Not reported	

Tsoutsoura 2023, Greece.	144 patients	Pain at the injection site: 73.4% (91/124)	Fatigue: 19.4% (24/124)<br><br>Fever: 18.0% (22/124)<br><br>Myalgia (muscle pain): 7.3% (9/124)<br><br>Headache: 4.8% (6/124)	Not reported	Not reported	
Tylicki 2022, Poland	BNT162b2 Group: 60 patients	Details not given	Details not given	Not reported	Not reported	
	mRNA-1273: 23 patients	Details not given	Details not given	Not reported	Not reported	
Vaiciuniene 2021, Lithuania	136 patients	Not reported	Not reported	6 out of 167 vaccinated patients (3.6%) had biopsy-proven rejection during 9-month follow-up.	Not reported	

Wijtvliet 2022, Belgium	mRNA-1273 Group in KTR: 42 patients ,	Not reported	Not reported	Not reported	Not reported	
	BNT162b2 Group in KTRs: 91 patients	Not reported	Not reported	Not reported	Not reported	
Yi 2021, USA	145 patients	Not reported	Not reported	Not reported	Not reported	
Zadok 2021, Israel	42 patients	Pain at the injection site: 30/42 (71.4%) Redness: 2/42 (4.8%)	Fatigue: 6/42 (14.3%) Arthralgia: 5/42 (11.9%) Myalgia: 4/42 (9.5%) Headache: 2/42 (4.8%) Systemic fever: 1/42 (2.4%)	Not reported	Not reported	
Rozen-Zvi 2021, Israel	308 patients	Not reported	Not reported	Not reported	Not reported	
Midtvedt 2021, Norway	141 patients,	Not reported	Not reported	Not reported	Not reported	

Midtvedt 2022, Norway	188 patients	Not reported	Not reported	Not reported	Not reported	
Miele 2021, Italy.	16 patients	Not reported	Not reported	Not reported	Not reported	
Ducloux, 2021, France	153 patients	Not reported	Not reported	Not reported	Not reported	
Erol 2021, Turkey	Sinovac subgroup: 31 patients	Arm pain: 6/31 (19.4%)	Fever: 3/48 (6.3%) Headache: 1/48 (2.1%) Tinnitus: 1/48 (2.1%) Fatigue: 5/48 (10.4%) Myalgia/Arthralgia: 3/48 (6.3%) Dizziness: 1/48 (2.1%)	Acute T-cell-mediated rejection in a liver transplant recipient, 1/48 (2.1%)	Not reported	
	BioNTech group: 17 patients	Arm pain: 13/17 (76.5%)			Not reported	
Haidar 2022 USA	183 patients	Not reported	Not reported	Not reported	Not reported	

Middleton 2021, United Kingdom	698 patients	Not reported	Not reported	Not reported	Not reported	
Mulder 2022. The Netherlands	476 patients	Not reported	Not reported	Not reported	Not reported	
Nazaruk 2021, Poland	61 KTRs	Not reported	Not reported	Not reported	Not reported	
	55 LTRs	Not reported	Not reported	Not reported	Not reported	
Correia 2022, Portugal	70 patients	Not reported	Not reported	Not reported	Not reported	
	61 patients	Not reported	Not reported	Not reported	Not reported	

Prendecki 2021, United Kingdom	Previous infection: 152 patients.	Not reported	Not reported	Not reported	Not reported	
	Infection naïve patients, BNT162b2 group: 410 patients.	Not reported	Not reported	Not reported	Not reported	
	Infection naïve patients, ChAdOx1 group: 358 patients.	Not reported	Not reported	Not reported	Not reported	
Saharia, 2022, United States	53 patients	Not reported	Not reported	Not reported	Not reported	
Sakai 2022, Japan	56 patients	Not reported	Not reported	Not reported	Not reported	

Spinner 2022, United States	40 patients	Not reported	Not reported	Myocarditis: 0/98 (0%) Antibody-Mediated Rejection (AMR): 2/40 (5%)	Not reported	
Strauss 2021, USA	161 patients	Not reported	Not reported	Not reported	Not reported	
Toniutto, 2022, Italy	Covid 19 naïve patients: 131 patients.	Not detailed	Not detailed	Not detailed	Not detailed	
	Covid 19 recovered patients: 12 patients.	Not detailed	Not detailed	Not detailed	Not detailed	
Yanis 2021, USA	56 SOT	Not detailed	Not detailed	Not detailed	Not detailed	

AE: Adverse Events; SAE: Serious Adverse Events; SOT: Solid Organ Transplant; KTR: Kidney Transplant Recipients; LTR: Lung Transplant Recipients; HTR: Heart Transplant Recipients; DSA: Donor-Specific Antibody; BPAR: Biopsy-Proven Acute Rejection; MMF/MPA: Mycophenolate Mofetil / Mycophenolic Acid; mRNA: Messenger RNA (referring to the vaccine platform, e.g.,

BNT162b2/Pfizer, mRNA-1273/Moderna); V: Vector (referring to viral vector vaccines, e.g., ChAdOx1/AstraZeneca, Ad26.COV2.S/Janssen).

Electronic Supplementary Figure 1. Forest plot for overall seroconversion.

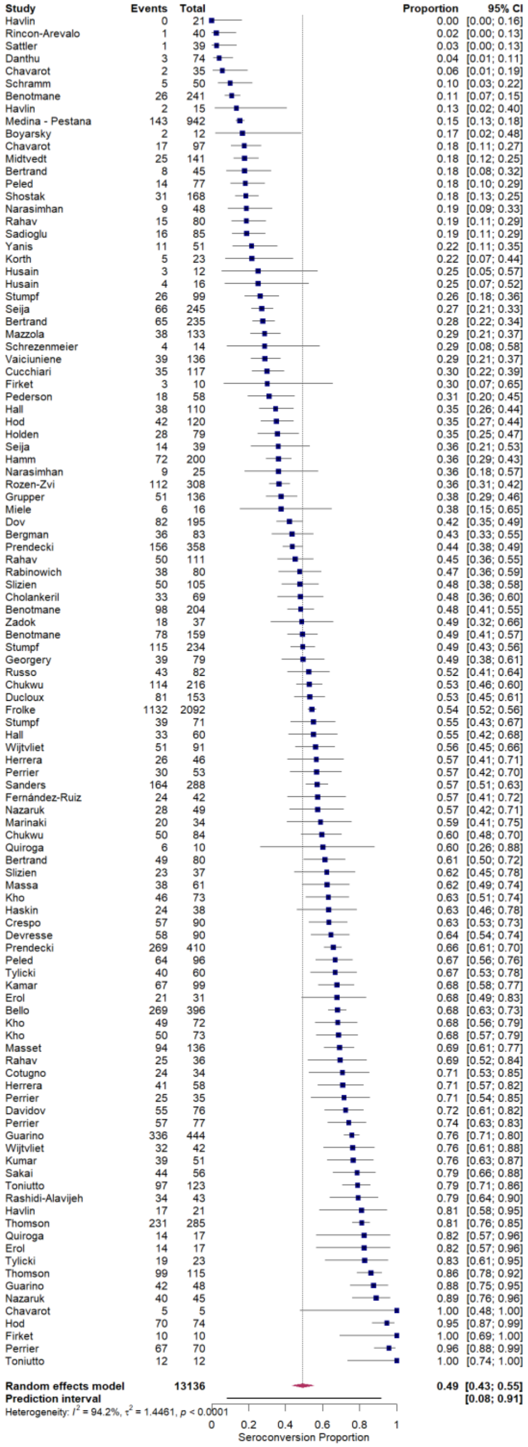

The plot displays the individual study proportions (squares) and their corresponding 95% confidence intervals (horizontal lines). The size of each square is proportional to the weight of the study in the meta-analysis. The diamond at the bottom represents the overall pooled estimate of seroconversion proportion and its 95% confidence interval. Total events and total participants are listed for each study.

Electronic Supplementary Figure 2. Forest plot for seroconversion with BNT162b2.

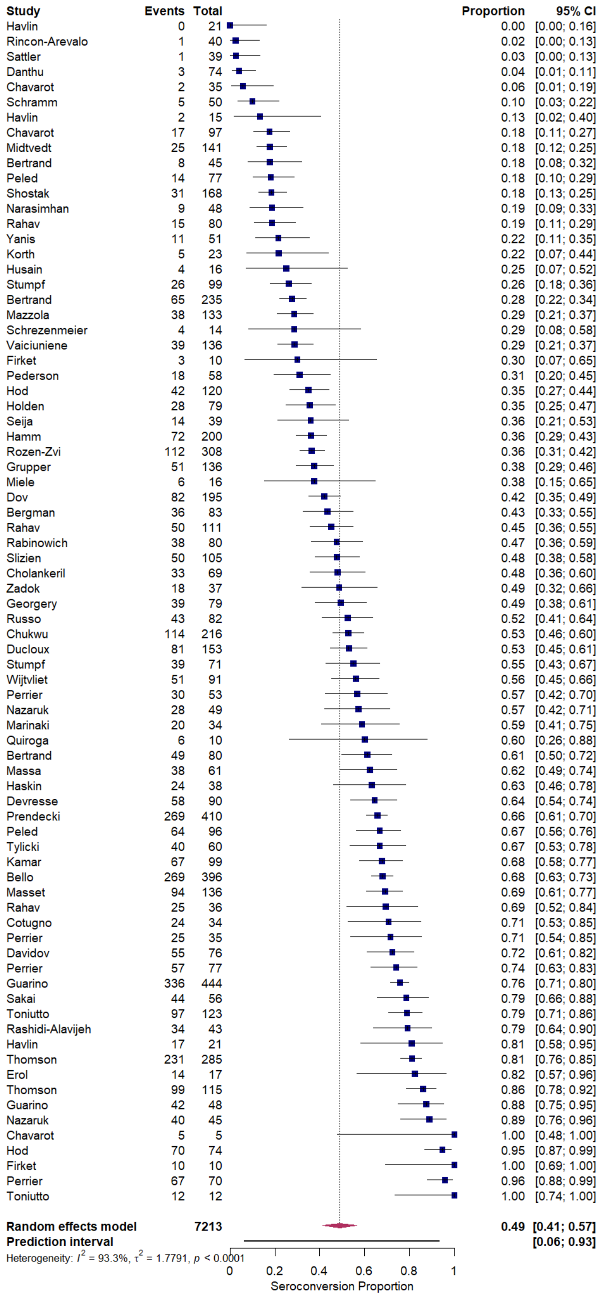

The plot displays the individual study proportions (squares) and their corresponding 95% confidence intervals (horizontal lines). The size of each square is proportional to the weight of the study in the meta-analysis. The diamond at the bottom represents the overall pooled estimate of seroconversion proportion and its 95% confidence interval. Total events and total participants are listed for each study.

Electronic Supplementary Figure 3. Forest plot for seroconversion in Kidney transplant recipients.

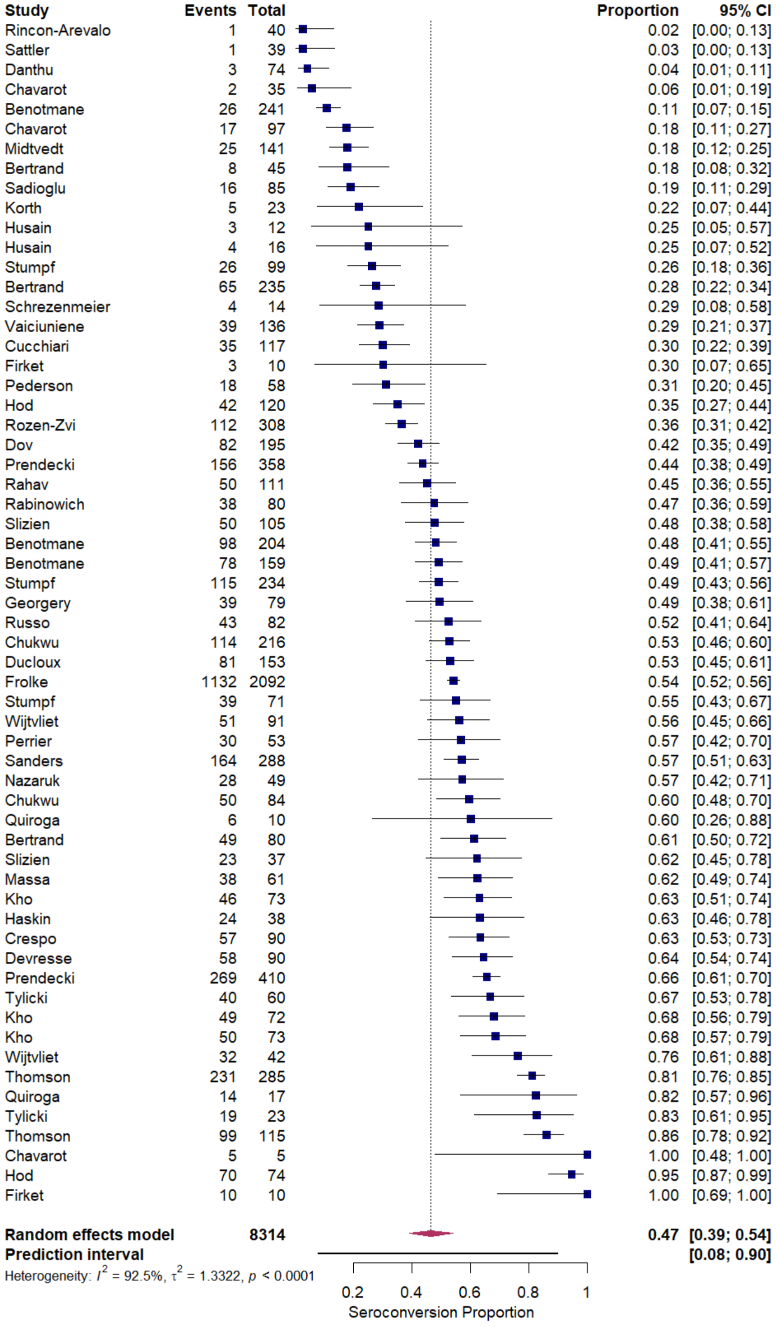

The plot displays the individual study proportions (squares) and their corresponding 95% confidence intervals (horizontal lines). The size of each square is proportional to the weight of the study in the meta-analysis. The diamond at the bottom represents the overall pooled estimate of seroconversion proportion and its 95% confidence interval. Total events and total participants are listed for each study.

Electronic Supplementary Figure 4. Forest plot for seroconversion amongst those without history of prior COVID-19 infection.

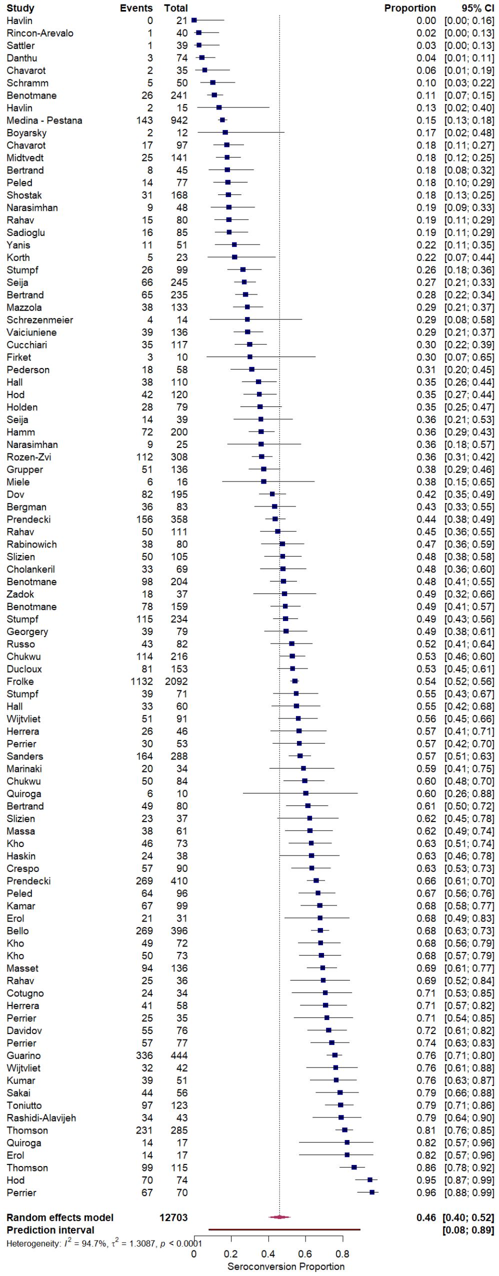

The plot displays the individual study proportions (squares) and their corresponding 95% confidence intervals (horizontal lines). The size of each square is proportional to the weight of the study in the meta-analysis. The diamond at the bottom represents the overall pooled estimate of seroconversion proportion and its 95% confidence interval. Total events and total participants are listed for each study.

Electronic Supplementary Figure 5. Forest plot for seroconversion with two doses of vaccine.

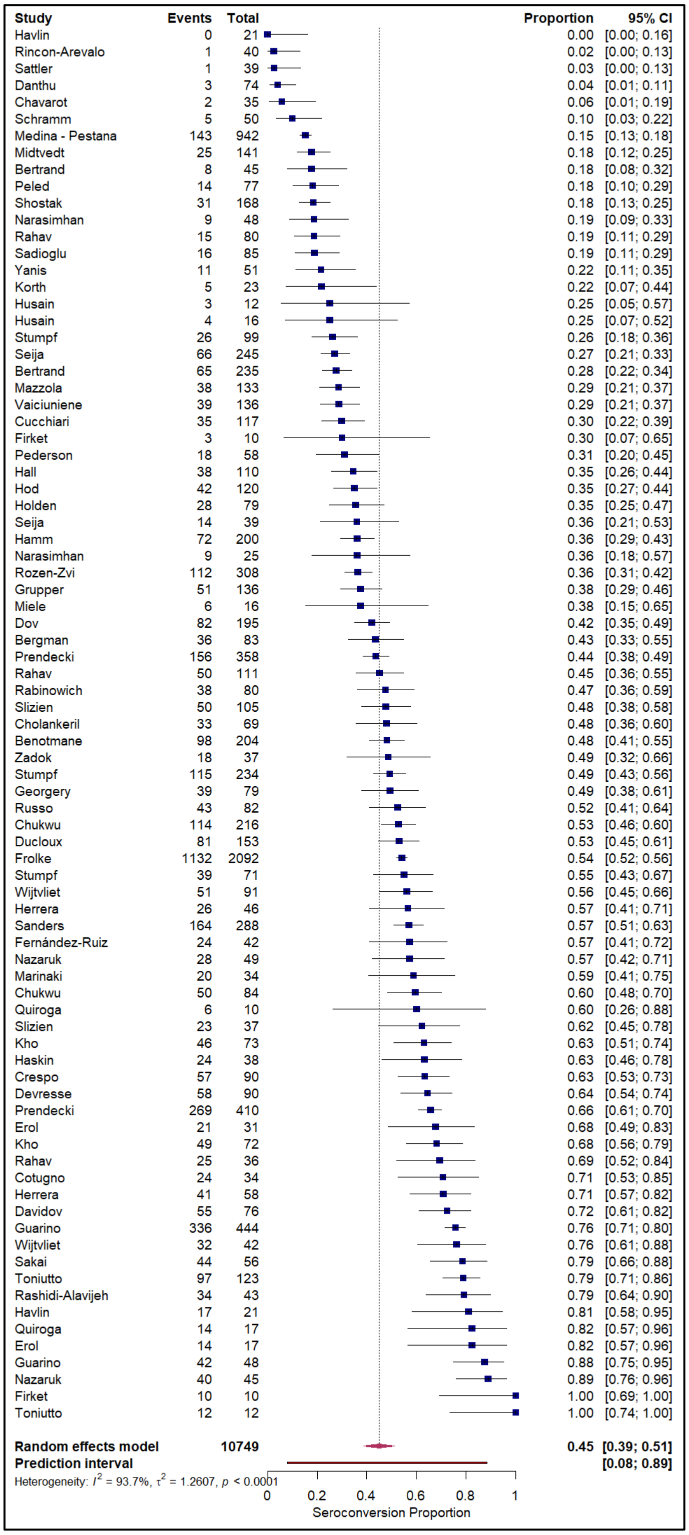

The plot displays the individual study proportions (squares) and their corresponding 95% confidence intervals (horizontal lines). The size of each square is proportional to the weight of the study in the meta-analysis. The diamond at the bottom represents the overall pooled estimate of

seroconversion proportion and its 95% confidence interval. Total events and total participants are listed for each study.
